# Revolutionizing the female reproductive system research using microfluidic chip platform

**DOI:** 10.1186/s12951-023-02258-7

**Published:** 2023-12-19

**Authors:** Jinfeng Yan, Tong Wu, Jinjin Zhang, Yueyue Gao, Jia-Min Wu, Shixuan Wang

**Affiliations:** 1 National Clinical Research Center for Obstetrical and Gynecological Diseases, Tongji Hospital, Tongji Medical College, Huazhong University of Science and Technology, Wuhan, China; 2 Key Laboratory of Cancer Invasion and Metastasis, Ministry of Education, Tongji Hospital, Tongji Medical College, Huazhong University of Science and Technology, Wuhan, China; 3 Department of Obstetrics and Gynecology, Tongji Hospital, Tongji Medical College, Huazhong University of Science and Technology, No. 1095, Jiefang Avenue, Wuhan, 430030 China; 4 State Key Laboratory of Materials Processing and Die & Mould Technology, School of Materials Science and Engineering, Huazhong University of Science and Technology, Wuhan, 430074 China; 5 Engineering Research Center of Ceramic Materials for Additive Manufacturing, Ministry of Education, Wuhan, 430074 China

**Keywords:** Female reproductive system, Women health, Microfluidic chip, Organ-on-chip

## Abstract

Comprehensively understanding the female reproductive system is crucial for safeguarding fertility and preventing diseases concerning women's health. With the capacity to simulate the intricate physio- and patho-conditions, and provide diagnostic platforms, microfluidic chips have fundamentally transformed the knowledge and management of female reproductive health, which will ultimately promote the development of more effective assisted reproductive technologies, treatments, and drug screening approaches. This review elucidates diverse microfluidic systems in mimicking the ovary, fallopian tube, uterus, placenta and cervix, and we delve into the culture of follicles and oocytes, gametes’ manipulation, cryopreservation, and permeability especially. We investigate the role of microfluidics in endometriosis and hysteromyoma, and explore their applications in ovarian cancer, endometrial cancer and cervical cancer. At last, the current status of assisted reproductive technology and integrated microfluidic devices are introduced briefly. Through delineating the multifarious advantages and challenges of the microfluidic technology, we chart a definitive course for future research in the woman health field. As the microfluidic technology continues to evolve and advance, it holds great promise for revolutionizing the diagnosis and treatment of female reproductive health issues, thus propelling us into a future where we can ultimately optimize the overall wellbeing and health of women everywhere.

**Graphical Abstract:**

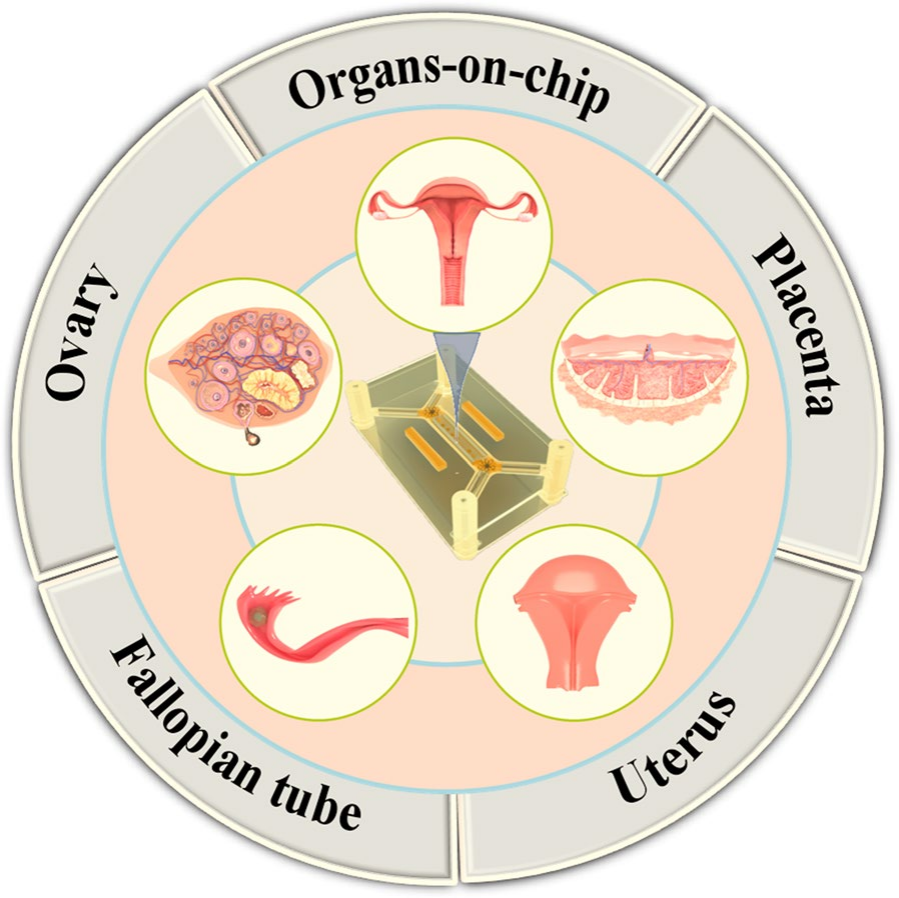

## Introduction

The reproductive health is strongly linked to a woman’s reproductive ability and quality of life, accounting for one-third of women’s health illness. The reproductive health problems are manifested by skin and body size, alteration, psychological and functional impairments, as well as emotional changes, such as insatiability, fear, and anxiety [1–5]. Some malignant diseases like cervical and ovarian cancer are even life-threatening [6]. Therefore, maintaining good reproductive system health is crucial for women. The female reproductive system (FRS) is a very complex physiologic system consisting of ovaries, fallopian tubes, uterus, cervix and vagina. These organs possess an intense interdependence and communicate through endocrine signals (i.e., hormones in the blood circulation), which is necessary for the achievement of the healthy function of the reproductive system [7]. Any of its disturbance will affect women’s fertility and lead to related diseases, including polycystic ovary syndrome, endometrial hyperplasia, endometriosis, pelvic organ prolapses, cancers, etc., whose incidence has increased over the past few years [8, 9]. Traditional treatments are primarily based on therapeutic drugs, and specially, surgery and chemotherapy are main strategies for gynecological tumors. Furthermore, secondary damage from chemotherapy can injure fertility function, leading to decreased ovarian reserve, ovarian insufficiency and even premature ovarian failure [10]. Consequently, it is urgent to find some effective treatments with few side effects to maintain the normal physiological function of women.

Tissue engineering is a multidisciplinary field that seeks to promote the functional and morphological reconstruction of tissues and organs after injury. Its components encompass seeding cells, biomaterial scaffolds, dynamic culture conditions, and micro-physiological environment simulations. By creating artificial organs, it can regenerate damaged or diseased tissue and restore normal organ function [11, 12], which provides a promising approach to addressing the limitations of current reproductive therapies and improving patient outcomes. The development of tissue engineering scaffolds, the application of microfluidic chip engineering technology, decellularized extracellular matrix [13]. Organoids approaches in treating premature ovarian failure, in vitro culture of follicles, uterine cavity adhesion, uterine defect repair, and pelvic organ prolapse are becoming increasingly widespread [14]. Compared with traditional treatment methods, tissue engineering can customize the treatment plan, according to the individual differences of patients, promote tissue regeneration and repair, improve the therapeutic effect and reduce the surgical trauma and recovery time [15]. It has the potential to revolutionize the management of reproductive diseases and improve current therapies as described above.

Microfluidic chips provide critical advantages over other tissue engineering methods. First of all, microfluidic chips transform the static characteristics of two-dimensional (2D) cell and tissue culture technology into more complex and dynamic in vitro platforms [16, 17]; Secondly, microfluidic chips require only a small number of cells, which allows researchers to collect valuable samples of limited size [18]; Thirdly, microfluidic models perform better in predicting therapeutic responses than traditional methods because they may simulate complex physiological microenvironment [19]; Last but not least, microfluidic chips can be integrated with digital platforms such as biological detection systems and sensors to achieve real-time detection [20, 21]. Therefore, the microfluidic chip provides a good platform for studying the FRS because it can reproduce the connections and microenvironment clues that greatly affect cellular structure, function, and growth between different reproductive organs and tissues [22]. Furthermore, the combination of microfluidic chips and assisted reproductive technology (ART) is also a current research hotspot. The microfluidic technologies can be combined with various ART procedures to achieve embryo and gamete (sperm and oocyte) analysis, classification, operation, culture and monitoring [23, 24], which will make up for some technical deficiencies of ART to assist in solving female fertility problems.

Although there have been reviews of microfluidic methods, and their application in the FRS [25–28]. Nevertheless, no paper has conducted a comprehensive study on the use of microfluidic chips. To the best of our knowledge, this is the first systematic and comprehensive exposition of the broad functions of microfluidic chips in the FRS. In this paper, we review the current status of microfluidic chips, the materials and methods used during fabrication, and applications in the FRS, including the simulation of female reproductive organs (ovary, fallopian tube, uterus, cervix) and related diseases. The latest advancement of microfluidic technology has realized the integration of on-chip sensors, actuators and micropumps to realize the integration of various microfluidics, which provides a powerful platform for ART. Finally, we summarize the latest development trends and challenges in this field. It provides a unique opportunity for the future development of the female reproductive disease treatment system.

## The development and application of microfluidic chips

Microfluidic chips consist of micro-scale channels and chambers with integrated seals, valves, and pumps for fluid manipulation, facilitating fluid handling, mixing, and separation [29, 30]. It initially engaged micro-electromechanical systems (MEMS) related technology to fabricate 2D patterns through lithography and mass production, typically employing crystalline silicon and glass as materials [31, 32]. However, with advances in technology, polymer materials are increasingly being used due to their wide range, vast number, cost-effectiveness, ease of processing, and scalability. The polymer materials used to fabricate microfluidic devices include thermoplastics (e.g., polyamide, polymethyl methacrylate, polycarbonate, and polyethylene terephthalate) [33, 34], curing type (like polydimethylsiloxane, epoxy resin and polyurethane) [35], and solvent evaporation type (such as acrylic acid, rubber and fluoroplastics) [36]. Among these, polydimethylsiloxane (PDMS) is especially popular in soft lithography techniques due to its capability to produce high-precision microstructures, elasticity, ability to integrate with external components, stable temperature gradients, permeability to visible and ultraviolet light, non-toxicity, and suitability for carrying out biological experiments [37]. In 1998, the Whitesides team first developed PDMS-based soft lithography technology, which quickly became a go-to method and revolutionized microfluidic prototype fabrication [38]. The soft lithography technology adopts photoresist as the material to achieve submicron-scale resolution and precise control, so that the exposure dose and time can be precisely tailored to meet desired requirements [39, 40]. However, despite the promising prospects, soft lithography entails significant investment costs, as the necessary equipment can be quite expensive [41]. Additionally, the preparation process is complex and demands a high degree of skill and experience, challenging the feasibility of large-scale chip fabrication. As the twenty-first century began, injection molding technology became a major method for mass-producing microfluidic chips [42], and has emerged as an efficient method for producing microfluidic chips in large batches. This technique involves the use of large injection molding machines, which enables the manufacture of microfluidic chips with complex structures, such as numerous small pores [43]. It should be noted that the internal structure of microchannels tends to be relatively simple. Moreover, injection molding is restricted to meltable polymers as the material of choice, which limits the range of available materials that can be used in the fabrication process [44]. Recently, 3D printing technology has also been proposed and adopted as a prototype design technique for microfluidic devices, which can print microfluidic chips with complex structures in a high-resolution, fast, customizable and one-step manner [45]. There have been numerous researches on fabricating of microfluidic chips by 3D printing [46–52], and we will focus on its future development.

Microfluidic chips have been extensively used in the field of biotechnology for various applications. Their major advantages are the ability to precisely control the biochemical and cellular environment to mimic the in vivo chemical gradients and biomechanical conditions. Most importantly, microfluidic chips can reproduce pivotal functions of specific organs and tissues. And they have been used to culture living cells in continuous perfusion and micron-sized chambers, which called organs-on-chips [53]. In comparison to traditional animal models, organs-on-chips are more human-related, while also significantly reducing time and costs. The optical characteristics of microfluidics further enable real-time imaging and detection of cell activity. Over the past decade, several organs-on-chips for tissue or organ models have been developed, such as for the lung [54–61], kidney [62–66], intestine [67–71], liver [72–77], heart [78–81], blood vessel [82–87], skin [88–91]. Furthermore, these microfluidic chips can be connected by a circulation system to form a human body-on-chip, thus creating a more physiologically relevant simulation [92], which is an important development direction of microfluidic chip research.

**Fig. 1:**
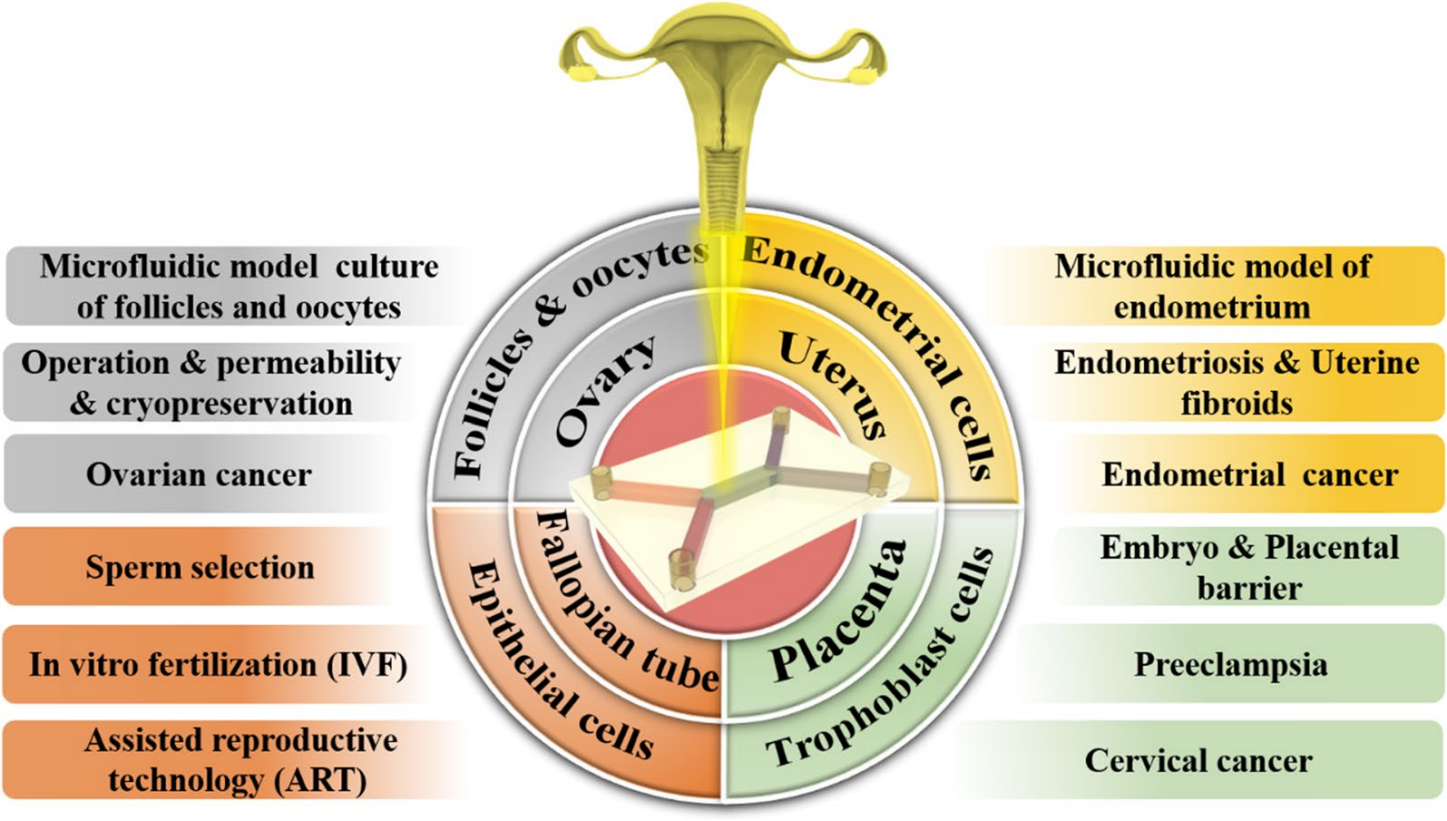
Schematic diagram of the microfluidic chip in the female reproductive system. It mainly includes microfluidic chips for the ovary, uterus, fallopian tube, placenta and their corresponding various cells and disease models

Microfluidic chips provide a unique opportunity to study the FRS. The application of microfluidic chips in the FRS began in the 1990s. At the earliest time, the birth of the first test-tube baby in Oldham, England, marked the beginning of ART in 1978 [93]. Since then, scientists have been striving to improve the method of in vitro fertilization by various means. Wang et al. [94] developed a new device in 1992 that enables the collection and selection of motile spermatozoa for use in ART such as intrauterine or in vitro fertilization. The device consists of a fluid-filled glass tube with a special design that directs the moving sperm to the upper arm of the tube, while other components such as dead or immotile spermatozoa, seminal fluid, and debris are left behind in the lower arm, thus allowing for the separation and selection of high-quality, motile spermatozoa for further use. Subsequently, Lih et al. [95] created a plexiglass microchamber with a loading well and a recessed side well. The device enriched sperms up to 13 times, making it suitable for intracytoplasmic sperm injection (ICSI). Furthermore, the microchamber allows for the placement of oocytes in the side wells, serving as a repository for these critical cells. This initiated the application of microfluidics for gamete separation and processing, fertilization, and embryo culture to study in vitro fertilization and simulate the related functions of the fallopian tube. At the beginning of the twenty-first century, researchers began to use microfluidic devices to mimic the FRS and diagnose reproductive health statuses, such as ovarian function assessment, implantation diagnosis, maternal antibody detection, and sexually transmitted disease detection [25]. It is also used to study the role of estrogen, analyze the causes of infertility, identify pathological changes that may lead to infertility, evaluate the potential of egg fertilization, and improve the efficacy of artificial fertilization [27]. As a result, microfluidics has become a new method for studying the menstrual cycle, pregnancy, female reproductive health and related diseases. The continuous development of microfluidic technology has enabled the related functions of FRS organs to be completely constructed in vitro, making it possible to develop microfluidic comprehensive diagnostic equipment for clinical use. Furthermore, the combination of whole genome sequencing technology and the microfluidic chip can enable doctors to gain a more accurate understanding of the pathogenesis of FRS diseases [96], thereby providing a better therapeutic strategy. Additionally, the development of new technologies for ART using microfluidic chips has been a hot topic for researchers. So, an in-depth understanding of the molecular, cellular and genetic mechanisms of the reproductive system using microfluidic chips will help develop more effective assisted reproductive technologies, treatment methods for reproductive aging, and screening drugs for female-specific diseases. By and large, microfluidic chip technology has established itself as a comprehensive solution across the spectrum of female reproductive health care, encompassing diagnostic procedures, fertility evaluations, adjunctive IVF technologies, drug testing, and disease modeling, etc., and the breadth of its application is a testament to its adaptability within the field. Nonetheless, the practical application of these chips is accompanied by a set of considerations that are critical to their successful implementation. These considerations have been systematically compiled in Table 1, providing a concise reference for current and prospective applications (see Fig. 1).

**Table 1:** Comparisons among various microfluidic chips designed for different parts of the female reproductive system

Microfluidic chip type	Application field	Reproductive organs	Compatibility with downstream analysis	Issues of note	Advantages
Diagnosis	STD testing, HPV screening	Cervix uteri and vagina	High	Limited specificity and sensitivity	Easy integration with portable devices
Fertility testing	Ovulation monitoring, Fertility health	Ovary	Low	Variations of menstrual cycle	Synchronization with patient's menstrual cycle required
IVF assistance	Oocyte capture, embryo culture	Fallopian tube	Low	Necessity to mimic the natural reproductive environment	Precise environmental control
Drug screening	Drug response assessment	All	High	Simulation of drug metabolism and physiological responses	High-throughput analysis
Disease modeling	Endometriosis, polycystic ovary syndrome cancer	All	High	Complexity in reproducing disease characteristics	High-fidelity tissue engineering
Self-management	Menstrual cycle tracking	Uterus and vagina	Low	User-friendliness and data privacy	Synchronization with smart devices
Detection	Hormone level monitoring	Ovary	High	Long-term stability and reliability	Micro-sample processing

*STD* Sexually transmitted diseases; *HPV* Human papillomavirus; *IVF* In-vitro fertilization

## Ovarian research using microfluidic systems

**Fig. 2:**
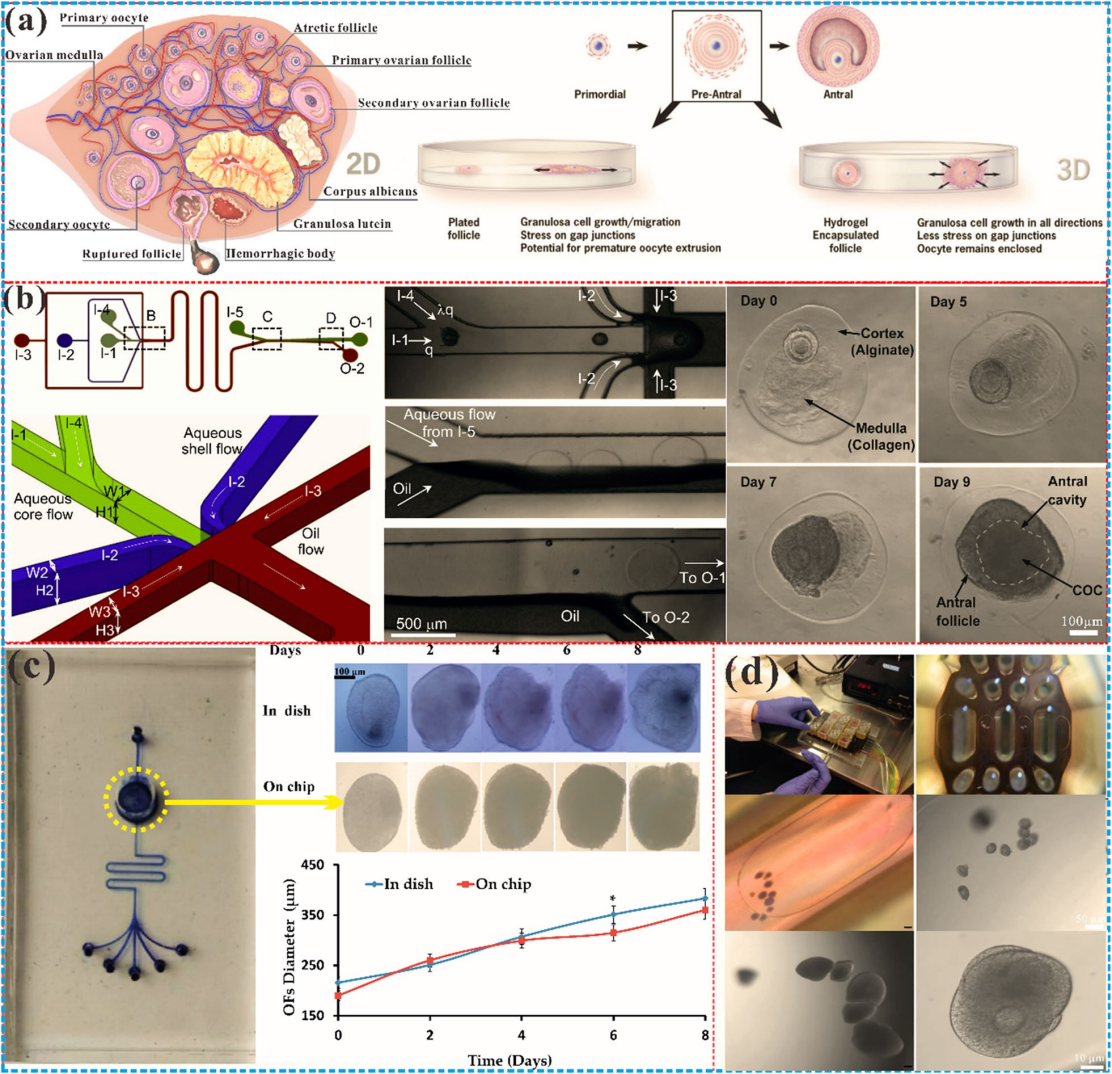
Microfluidic chips for ovarian follicles. **a** The ovary is composed of the reproductive epithelium, tunica albuginea, cortex, and medulla. Among them, the cortex is the outer layer of the ovary, including follicles, mesenchyme and antral cavity. The medulla is the inner region of the ovary, which is composed of connective tissue, blood vessels, nerves and lymphatic vessels. The 3D culture system is the most suitable platform for in vitro culture of follicles and oocytes. Adapted with permission from Ref. [109]. Copyright 2010 Springer Nature. **b** A microfluidic flow-focusing device is used to encapsulate early secondary preantral follicles in core–shell microcapsules to produce biomimetic ovarian microtissue [121]. Copyright 2014, Elsevier. **c** Cultured ovarian follicles in a dish and on-chip, and the two results are consistent, indicating that microfluidics can replace traditional culture systems. Reproduced with permission from Ref. [124]. Copyright 2017 MDPI. **d** A multi-unit microfluidic platforms supports to research the endocrine, immune and metabolic factors involved in the development of follicles [123]. Copyright 2017 Springer Nature

The ovary is a fundamental reproductive organ and can be divided into four regions: the surface epithelium, tunica albuginea, cortex, and medulla [97], which is shown on the left of Fig. 2a. The follicles are the principal functional units, and made up of oocytes encompassed by granulosa cells. They mature and develop through bidirectional signaling interactions with the extracellular matrix and growth factors, guided by critical feedback loops [98]. The ovary is responsible for ovulation and secretion of essential hormones, estrogen, and progesterone, which regulate the menstrual cycle, pregnancy, lactation in women, secondary sexual characteristics, mood, and bone mineral density [99, 100]. Unfortunately, the ovary may succumb to various diseases such as cysts, tumors, or aging. Cysts are fluid or solid-filled sacs forming in the ovary that may be benign but could also result in malignancy or complications [101, 102]. Tumors denote anomalous tissue growth within the ovary and can be benign or malignant, with the latter being a prevalent form of gynecological cancer [103]. Ovarian aging signifies the decline in ovarian function in the female reproductive system, leading to abnormal reproductive cycles, disorders, and ultimately infertility, adversely affecting women's health.

In recent years, researchers have sought to leverage the potential of microfluidic technology in the field of ovarian research, to advance our understanding of ovarian physiology and pathology, and facilitate the development of novel therapeutic interventions for ovarian disorders [104]. The unique capacity of microfluidic technology to replicate complex cellular microenvironments with precision is of particular importance in the examination of ovarian diseases. By providing multiple experimental conditions and varying experimental parameters, microfluidic chips offer an accurate simulation of the intricate cellular processes in the ovary beyond traditional model parameters. Microfluidic technology also can assist in comprehending the mechanisms of ovarian disorders, which is essential for the development of more effective treatment of ovarian diseases. Furthermore, microfluidic technology can be utilized to monitor and control drug delivery, leading to greater treatment efficacy [105]. With its potential for clinical application in ovarian research, microfluidic chip technology holds promise in driving new discoveries and innovations in the field.

### Microfluidic chips for follicles

#### Conversion from 2 to 3D

The initial studies on follicle culture in vitro predominantly employed traditional 2D culture methods. This approach was executed in various configurations, such as using porous plates, cell culture dishes, or coverslips coated with extracellular matrix (ECM) proteins such as collagen, fibronectin or laminin [106, 107]. Preantral follicles grown in porous plates or micro drop cultures have been shown to produce mature oocytes (Fig. 2a on the right) [108, 109]. Eppig and Schroeder have developed a new method, which was able to mature mouse preantral follicles in vitro using collagen-impregnated gels, and led to live births following in vitro fertilization of oocytes [110]. The same collagen culture method was also able to mature primordial follicles and produce living offspring when in situ cultured in newborn mouse ovaries for eight days [111]. Nonetheless, the 2D culture systems destroy the three-dimensional structure of follicles, resulting in somatic cells migrating to the bottom of culture wells and thereby losing contact with oocytes. This is a departure from the in vivo microenvironment, where cells and tissues interact intricately with an array of adherent ligands within 3D ECM [112]. Since cell–cell and cell–matrix interactions are critical to the follicle and oocyte development [113]. 3D follicular culture systems have shown better rates of follicular survival and growth than their 2D counterparts, while preserving follicular morphology and reducing oocyte maturation gene expression to better mimic in vivo conditions.

The 3D culture of follicular cells is essential for studying follicular development and function. However, choosing the appropriate 3D culture system is a challenging task due to its respective advantages and disadvantages. For instance, the 3D floating culture system preserves the natural morphology and function of follicles but may promote follicular adhesion, reducing nutrient and oxygen diffusion [114]. In contrast, the 3D embedding culture system provides a microenvironment similar to that of in vivo but may affect the growth and differentiation of follicles [115]. Similarly, the 3D printing system allows customization of the microenvironment but may damage the follicles or limit the selection of materials or parameters [116]. Moreover, the culture environment must provide sufficient nutrition and oxygen while maintaining suitable temperature, pH, and hormone levels [117]. The fluid flow mimicking the ovary environment is also critical for optimal follicular development. The culture conditions should be adjusted according to the follicular developmental stages to provide the best results. In conclusion, a systematic comparison and evaluation of different 3D culture systems are necessary to determine the optimal conditions for follicular culture in vitro.

#### Follicles cultured in active dynamic conditions

The microfluidic chip boasts a remarkable ability to emulate the micro-physiological milieu, propelling the in vitro cultivation of follicular cells under tiny hydrodynamic conditions. Recent research has established that the microfluidic culture system significantly heightens the production of intracellular micro active substances, amplifies cellular proliferation and apoptosis, and instigates the transduction of extracellular signals [118, 119]. As such, this cutting-edge technology presents an effective means of regulating the advancement and differentiation of follicles. The microfluidic technology facilitates the management of liquid flow in minuscule tubes, thus enabling the cultivation of multiple follicles concurrently while accurately manipulating and regulating the liquid [120]. Alginate and collagen were used to create ovarian cortex and medulla tissue for microfluidic generation of biomimetic ovarian microtissues for a micro three-dimensional 3D culture of early preantral follicles [121], as shown in Fig. 2b. Notably, microfluidic technology optimizes cells’ responses to environmental shifts such as antiviral and anti-inflammatory [122].

The delicate process of culturing follicular cells in three-dimensional space can be achieved through the use of hydrogel microspheres encapsulated in a microfluidic chip. This innovative method serves to maintain the biological activity and function of follicles, while also allowing for real-time monitoring of their physiological parameters [119]. The microfluidic chips enable precise control and operation of a single follicle, as well as facilitate interactions and communication between multiple follicles [123]. Recent studies have demonstrated the efficacy of microfluidic chips in the culturing of single human preantral follicles, with results comparable to those obtained through traditional methods [124], which is shown in Fig. 2c Furthermore, the application of this technology in constructing double-layer follicles composed of mouse granulosa cells has yielded promising results, with the resulting follicles exhibiting endocrine function such as the production of estradiol, progesterone, and androstenedione [125]. Microfluidic chips also have the potential to be integrated with other human organ chips to construct an integrated human body model, so as to better study the endocrine, immune, metabolic and other factors involved in follicular development [123], as shown in Fig. 2d. It can be seen that microfluidic culture can effectively improve the proliferation, differentiation and apoptosis of follicular cells, thus providing important technical support for the study of stem cell therapy and providing a novel and efficient platform for the in vitro culture of follicular cells.

The development of microfluidic chips for follicular cell culture poses significant challenges, as it necessitates precise control over microchannel size, shape, and material to accommodate different stages of follicular growth. Such chips must enable real-time monitoring and assessment of the activity, maturity, and quality of follicular cells, along with their interactions with other cells or medications. As no existing approach is capable of simultaneously satisfying all these conditions, ongoing optimization of microfluidic chip materials, structures, parameters, and operating conditions is necessary to enhance their multifunctionality and reliability.

### Microfluidic platforms for oocyte

Oocytes are essential cells capable of developing into eggs, which can be matured either in vivo or in vitro. These cells progress through three distinct stages: primary oocytes, secondary oocytes and mature oocytes [126]. Primary oocytes, which are diploid cells in the initial stage of meiosis, contain a substantial amount of nutrients and mRNA. As they move forward into secondary oocytes, they become haploid cells after the first meiosis and contain fewer nutrients and mRNA [127]. Finally, mature eggs undergo the second meiosis and become fully functional haploid cells capable of fertilization. In terms of in vitro culture, each stage of oocyte development requires a unique set of specific culture media and conditions. Primary oocytes benefit most from culture medium containing gonadotropins, growth factors, and antioxidants to foster their maturation process [128]. To maintain the viability and function of secondary oocytes, the culture medium requires substances that support their metabolic activity and gene expression. For mature eggs, the culture medium must protect their chromosome stability and nucleo-cytoplasmic interaction to effectively improve fertilization rates and developmental potential [129]. However, in vitro culture of oocytes is not without challenges, as metabolomics and proteomics may shift during these processes [130, 131]. Moreover, oocytes can only maintain their optimal state within a restricted time frame in vitro [132]. Therefore, appropriate methods are also needed to fill the in vitro model construction and cell culture of oocytes. Typically, the collection of immature oocytes involves the removal of granulosa cells and the screening of healthy oocytes. Traditional methods for oocyte retrieval involve the slicing or needle aspiration of ovarian tissue, followed by treating the tissue fragments in a solution containing digestive enzymes and mechanical oscillation to release primary oocytes [133]. Recently, microfluidic chips have shown promising results in combining chemical digestion and mechanical oscillations to remove cumulus cells in tissue fragments containing primary follicles to maintain the integrity and activity of primary oocytes [134]. Removing cumulus cells also facilitates the analysis of oocyte morphology and increases the success rate of subsequent procedures such as fertilization or nuclear transfer.

#### Optimization of oocyte screening

**Fig. 3:**
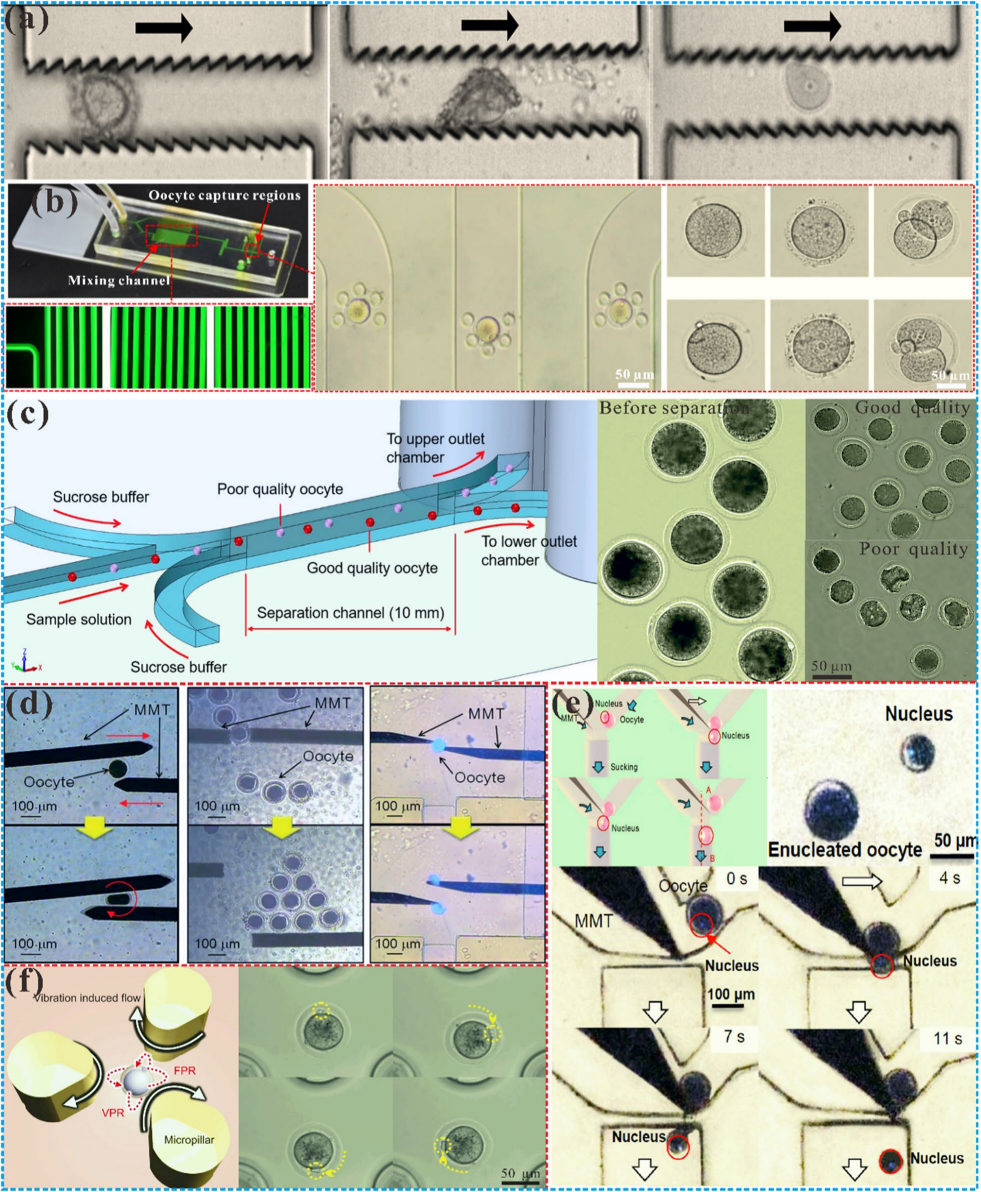
Microfluidic technology for oocytes. **a** Denuded oocytes were obtained by stripping cumulus cells in a microfluidic chip [140]. Copyright 2018 Royal Society of Chemistry. b Oocyte culture on a microfluidic chip. By utilizing multi-channel microfluidic chips and designing new microcolumns, which can now achieve reliable and consistent capture of viable oocytes and facilitate dynamic cell culture. [141]. Copyright 2020 Springer Nature. c A simple microfluidic method for separating high-quality oocytes from poor-quality ones based on the differential deposition rate in sucrose buffer [143]. Copyright 2018 Springer Nature. **d** The on-chip robots driven by the permanent magnet in microfluidic chip achieve the operation of oocytes [152]. Copyright 2011 Royal Society of Chemistry. **e** A micro-robot was used to achieve enucleation in the closed space of the microfluidic chip [157]. Copyright 2013 MDPI. **f** On-chip three-dimensional cell rotation method based on the vibration-induced flow [160]. Copyright 2015 Springer Nature

Cumulus cells are cubic cells that enclose egg cells and form a cumulus-oocyte complex (COC). In the process of artificial insemination, such as intracytoplasmic ICSI, cumulus cells need to be removed to allow sperm to enter the egg cells smoothly [135]. Conventional methods of removing cumulus cells include physical techniques such as blowing, sucking, or vibrating [136], as well as chemical methods using hyaluronidase enzymes [137]. However, such techniques may damage the egg cells and reduce their quality. Microfluidic chip technology offers a promising approach for the efficient and low-damage removal of cumulus cells in tiny fluid channels. For example, Zeringue et al. [138] utilized microchannels with optimized widths to apply negative pressure and gently detach cumulus cells from the COC while preserving the integrity of the egg cells [139]. Similarly, Weng et al. [140] developed a serrated-surface microfluidic device to detach cumulus-coronal cell clusters from oocytes, enabling the evaluation of oocyte quality and subsequent procedures such as in vitro fertilization (Fig. 3a). Compared with the mechanical transfer, the oocytes removed by this device have comparable fertilization and development ability. Figure 3b displayed a multi-channel microfluidic chip designed to provide repeatable oocyte capture and dynamic culture in screening, enabling simultaneous and non-destructive measurement of the permeability of multiple oocytes [141]. Ultimately, it is necessary to screen out good-quality oocytes for in vitro fertilization and other operations to ensure that the final in vitro fertilization results are not affected by poor oocytes.

Currently, the assessment of healthy oocytes relies heavily on subjective morphological criteria, limiting the accuracy of the screening process. To overcome this issue, novel oocyte sorting techniques have emerged involving microfluidic devices. These systems can utilize various physical and chemical parameters, such as size, deformability and chemical properties, to screen oocytes. For example, Nakahara et al. [142] developed an integrated microfluidic chip that employs automated evaluation of oocyte mechanical properties to sort oocytes, enhancing objectivity in the screening process. However, this approach relies on costly technologies, such as MEMS devices and control units. Alternatively, a simple and efficient method as shown in Fig. 3c involves the use of microfluidic devices based on differences in the sedimentation rate of oocytes in a sucrose buffer, which can isolate the high-quality oocytes according to the difference of the sedimentation rate [143]. Other techniques, such as microwell arrays and forward dielectrophoresis, have also proved effective in capturing and screening high-quality oocytes [144]. Despite these advances, the stability of microfluidic devices during cell transport and potential blockage and contamination within microchannels need to be further addressed [145]. Overall, microfluidic devices represent a promising platform to improve the efficiency and quality of oocyte screening in reproductive medicine research.

#### Estimation of oocyte permeability

Oocyte permeability refers to the ability of the oocyte membrane to pass water and other small molecules, which can be measured by water conductivity (Lp) and solute permeability (Ps). The permeability of the oocyte membrane changes with its maturation and development stages, which affects the survival rate of oocytes during cryopreservation [146]. The molecular channels on the oocyte membrane can regulate the osmotic pressure balance inside and outside the cell during the freezing, protecting the oocyte from damage thawing process [147]. Researching the permeability of oocyte membranes is of great significance for improving the success rate of ART, such as artificial insemination, in vitro fertilization and transplantation. The traditional method is to quantify the permeability parameters of oocyte membranes by microtubule perfusion and direct microscopic observation, and it cannot precisely measure the permeability of oocytes. Zhao's team recently developed a novel sandwich-structured microfluidic perfusion method to accurately characterize the permeability of human oocytes and achieve high-quality oocyte selection [148]. Whereas, the above method can only measure a single oocyte or a single cryoprotectant (CPA) concentration at a time and the measured oocyte development potential has not been fully verified. To overcome this limitation, a novel microfluidic chip was designed to measure the permeability of multiple oocytes simultaneously, providing a fast and efficient method for analyzing the membrane's properties [149]. Chen et al. [141] exploited a multi-channel microfluidic chip with a newly designed microcolumn, which can provide repeatable oocyte capture, and simultaneously measure the penetration reaction of three oocytes under different or the same concentration of CPA, greatly improving the measurement efficiency. Additionally, the chip's incorporation of a precise local temperature heater enabled the measurement of temperature-dependent membrane permeability, which plays an integral role in oocyte cryopreservation success [150]. Overall, these innovative microfluidic chip approach offers a non-destructive and efficient method for analyzing oocyte permeability, paving the way for improved reproductive medicine and pharmacology.

#### Manipulation of follicles and oocytes

The application of cloning technology in the field of assisted reproduction holds promise for the development of reproductive organ transplantation and genetically similar experimental animals. The cloning technology involves the steps of cell enucleation, donor cell injection, fusion of original oocyte and injection, which is carried out by using a glass capillary connected to a mechanical micromanipulator as an end effector [151]. Nevertheless, the traditional cloning process is complex and reliant on the operator’s skill level, which can limit its success rate, repeatability, and productivity. To widely popularize the application of cloning technology in the reproductive system, microfluidic chips utilizing magnetically driven microtools (MMT) have attracted significant research interest for oocyte operation [152–154], as shown in Fig. 3d. Masaya et al. [155] have utilized these chips to achieve precise cell manipulation and enucleation of porcine and bovine oocytes, the accuracy of the microfluidic denuclearization platform with disturbance observer PI level feedback control is improved by 16 times. One study even developed a micro robot capable of enucleating bovine oocytes on a microfluidic chip using external magnetic force, resulting in spherical enucleated oocytes with intact cell membranes [156]. And Feng et al. [157] realized the enucleation of bovine oocytes on the chip by using microrobot-assisted flow control in Fig. 3e, and constructed a single oocyte precise distribution system [158]. These advancements hold promise for expanding the application of cloning technology in assisted reproduction.

Moreover, microfluidic chips have emerged as a powerful tool in the field of cloning technology and ART, facilitating the delivery of exogenous molecules and improving the success rate of fertilization. An innovative approach utilizing micro-robotics and acoustic microfluidics to selectively rotate and direct the movement of oocytes, allowing for improved cutting accuracy and success rates [159]. Through injection, exogenous molecules can be delivered into recipient cells, thereby changing or regulating the gene expression and function of recipient oocytes. More importantly, enucleation and microinjection improve the success rate of fertilization and the characteristics of oocytes, involving the three-dimensional rotation of mammalian oocytes. And the posture or orientation of oocytes is pivotal in improving the success rate of oocyte enucleation and studying the characteristics of oocytes. Hayakawa et al. realized the directional control of oocytes on the chip driven by a vibration-induced flow. Compared with conventional work, the microfluidic rotation control has great advantages in control accuracy and speed, as shown in Fig. 3f [160]. It can well control the orientation of oocytes and improve the cutting accuracy of oocytes by adjusting the orientation and position of oocytes. A new method for realizing cell rotation in the chip by acoustic microfluidics generated by oscillating asymmetric microstructures, which can effectively regulate the rotation speed and direction of porcine oocytes, to conduct further research and operation on oocytes [161]. These novel techniques hold great promise for the advancement of ART, but further development of simplified and multi-functional microfluidic platforms is necessary for greater accessibility and utility.

### Applications in cryopreservation

Cryopreservation of ovarian tissue is a crucial method for safeguarding female fertility, particularly for those encountering ovarian dysfunction or loss due to treatment. Standard approaches such as cryopreservation of embryos, oocytes or ovarian cortical tissue have been complicated by osmotic, toxic, and ice crystal damage to the cells, hindering the improvement of cell quality. To address this, microfluidic chips offer a closed, controlled environment that can extend in vitro culture time and prevent exposure to harmful pollutants. In a recent study, Julieta et al. [162] utilized a microfluidic platform to culture cryopreserved human ovarian cortical tissue for 8 days, evaluating the impact of different flow rates on follicular activation and development. The dynamic system showed potential in promoting follicle transformation from the primitive stage to the second stage, albeit with low efficiency. As such, further optimization is required to enhance the efficacy of early follicular development in vitro.

The cryopreservation of oocytes in assisted reproduction relies on cryoprotectants, which can influence the survival, pregnancy, and fertilization rates of preserved cells. However, the use of cryoprotectants may also damage the organelles and ultrastructures of sensitive oocytes, leading to low embryo and delivery rates [163]. Recently, microfluidic chips have emerged as a promising technology to optimize cryopreservation by ensuring the integrity of oocyte morphology and function while achieving large-scale cell preservation and operation. Specifically, microfluidic devices were developed to quantify oocyte bodies in various cryoprotectants loading schemes and to create a precisely controlled continuous cryoprotectant profile for oocyte addition, enabling the reduction of the additional time to less than 15 min and minimizing the change in cell volume to less than 10% [164]. Additionally, microfluidic devices were used to remove cryoprotectants from blood samples, demonstrating the feasibility of this technology for cryoprotectant removal [165]. In fact, the addition and removal processes of cryoprotectants are related, and the selection of thaw solution concentration and equilibrium time depends on the concentration and equilibrium time of cells in the cryoprotectant. Therefore, it is necessary to use the advantages of microfluidic chips to further optimize the various processes of cryopreservation, so as to achieve the consistency of oocytes after cryopreservation with previous levels.

Two of the most commonly utilized cryopreservation methods for embryos are slow freezing and vitrification [166]. Intracellular ice crystal formation during freezing is a significant cause of cell damage, a challenge that both techniques aim to minimize. Cryoprotectants are used to reduce the formation of ice crystals, but excessive exposure to these agents can result in toxicity. And precision equipment and skilled operators are necessary to optimize freezing parameters, such as freezing rate [167]. Microfluidic technology offers new opportunities in the field of automated embryo processing and vitrification due to its unique capabilities. Its permits the testing of cryoprotectant concentrations during cryopreservation, and offers the possibility of automating the vitrification process [168]. Automation can reduce labor costs and experimentally facilitate protocol optimization. The critical point lies in the fact that automating the vitrification process is to replicate the washing and timing steps of a given scheme while maintaining complete control of the embryo. The droplets on the microfluidic platform can be used as microvess4aels to move embryos and place them in a series of different concentrations of cryoprotectants according to the requirements of the in vitro fertilization vitrification procedure. Pyne et al. [169] developed a digital microfluidic device that uses automated methods to obtain embryo survival and development rates comparable to manual operations. Compared with manual operation and channel-based microfluidic vitrification, the advantages of this method include automated operation, low-temperature protective agent concentration gradient generation, and the feasibility of loading and removing embryos. An independent microfluidic system was introduced to automate the CPA loading process with automated manual processes, and to break through the boundaries of low-temperature storage by minimizing osmotic stress, shortening the entire process and reducing molecular footprints [170]. Analogously, another open microfluidic chip system has been developed to adapt to embryo adaptation in the automatic pretreatment process of cryoprotective agents, resulting in a 100% success rate for automated embryo transfer and vitrification, with comparable embryo survival and development rates to manual operation [171]. Microfluidic automation is a promising area for the development of new vitrification protocols and their implementation in clinical in vitro fertilization (IVF) practice.

## Fallopian tube/oviduct-on-chips

The fallopian tube is a crucial anatomical structure that fulfills several fundamental functions during early pregnancy. It provides a suitable microenvironment for fertilization, embryonic development, and the transport of developing embryos to the uterus [172]. The fallopian tube is composed of multiple segments, each exhibiting unique physiological properties that facilitate the successful development of the embryos. At the ampullary-isthmic junction of the fallopian tube, the zygote is formed and subsequently transported to the uterine cavity, aided by various factors such as ciliated epithelium, smooth muscle, and oviductal secretion [173–176]. Moreover, the fallopian tube secretes several critical cytokines that support embryonic development and implantation [177]. However, due to the complex anatomical location and biochemical composition of the fallopian tube, there is a notable paucity of viable in vitro models that accurately mimic its function.

The fallopian tube plays a pivotal role in facilitating gamete interaction, fertilization, and early embryonic development. The function of fallopian tube epithelial cells (OEC) has been examined in traditional tissue culture dishes, but under these two-dimensional conditions, the OEC characteristics of cell differentiation are rapidly lost, such as collaboration and secretory activity [178]. The traditional two-dimensional tissue culture models fail to accurately mimic OEC differentiation and secretory activity. Bart Leemans et al. [179] have exploited a microfluidic chip-based culture model that supports the growth of differentiated and polarized horse OECs (EOECs). Their protocol involves indirect inoculation and the use of estrogen and progesterone-containing media to promote EOECs differentiation and polarization, resulting in a continuous monolayer characterized by the presence of cilia, microvilli, and tight junctions. The EOECs in the model secrete liquid and proteins while attracting sperm chemically. The researchers also observed that the EOECs monolayers could maintain their structure and function even under dynamic flow conditions. By developing this well-functioning equine oviduct model, the researchers provided a valuable tool for studying the physiology and pathology of fallopian tubes and other animal models. This work is particularly significant because few existing microfluidic chip models simulate the interaction between sperm and OECs in the fallopian tube.

**Fig. 4:**
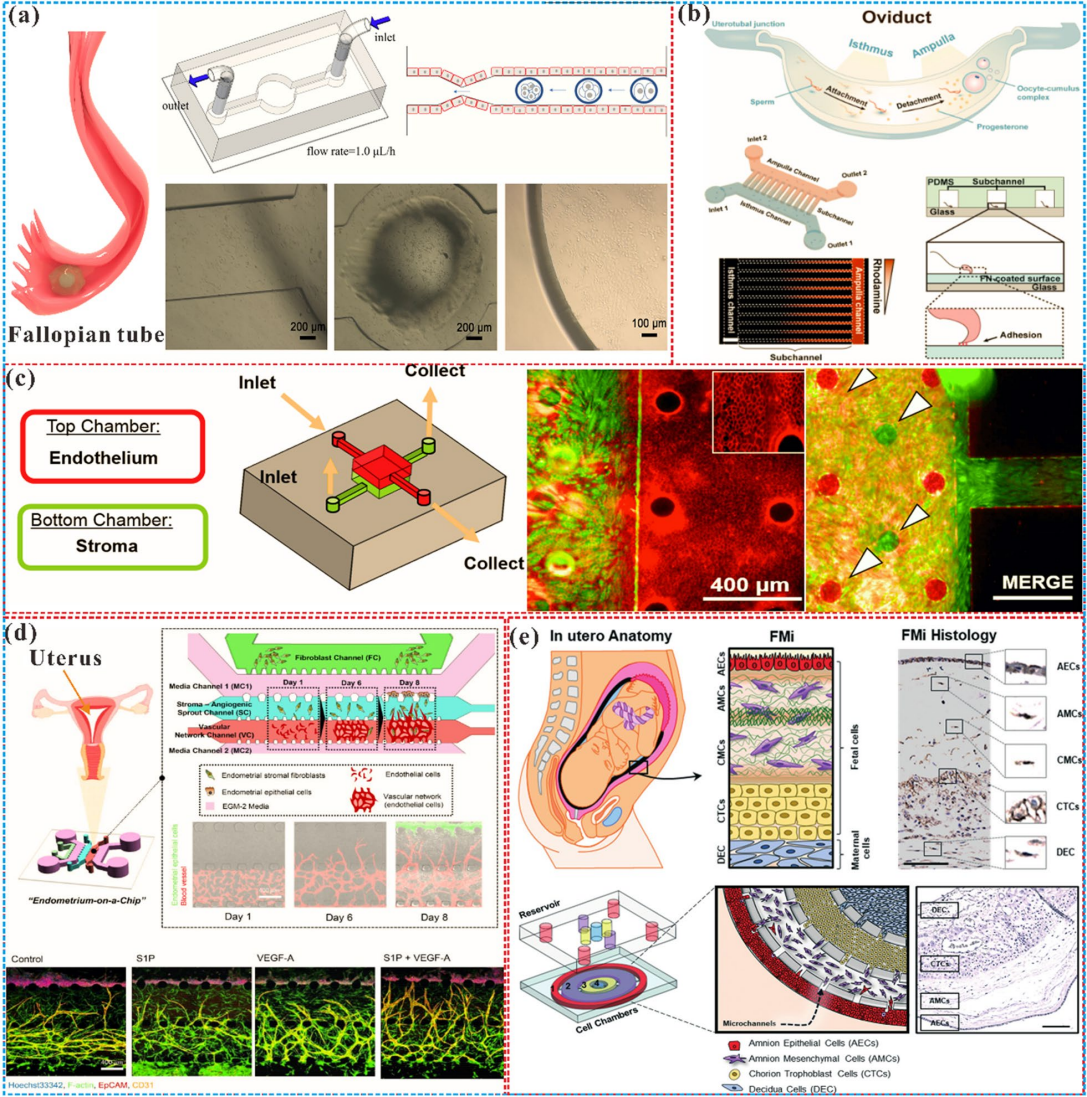
The microfluidic chips for fallopian tubes and uterus. **a** Microfluidic simulation of the fallopian tube, which established a tubal model on a chip to simulate in vivo embryo culture conditions. This novel on-chip fallopian tube model can optimize embryo culture conditions by reducing intracellular ROS levels. Reprinted with permission from Ref. [185]. Copyright (2022) Elsevier. **b** A microfluidic device was used to construct fallopian tube epithelial cells to study the behavior of sperm escaping from the fallopian tube surface [189]. Copyright 2023 American Chemical Society. c Long-term in vitro co-culture of human endometrial stroma and endothelial cells on microfluidics [195]. Copyright 2017 SpringerLink. d An organ chip using human endometrial epithelial cells, stromal fibroblasts, and endothelial cells to closely reproduce the endometrial microenvironment [197]. Copyright 2021 Oxford University Press. e A microfluidic chip was engaged to connect amniotic and chorionic mesenchymal cells, as well as chorionic trophoblast cells and decidual cells, researcher found that fetal exosome-mediated paracrine signals can cause inflammation and induce labor [198]. Copyright 2021 Royal Society of Chemistry

Several studies have demonstrated the potential of microfluidic technology in improving sperm selection for IVF and optimizing embryo culture conditions [180, 181]. The utilization of microfluidics has been found to improve the embryo fertilization rate [182] and blastocyst formation rate [183]. A dielectrophoresis microfluidic system simulates the oviduct of mammals through microchannels to achieve in vitro fertilization of imprinted control region (ICR) mice [184]. This method can increase the concentration of sperm near the oocyte and increase the probability of natural fertilization. Wang et al. established a tubal model on a chip to simulate in vivo embryo culture conditions [185], as shown in Fig. 4a, resulting suggests that this novel on-chip fallopian tube model can optimize embryo culture conditions by reducing intracellular reactive oxygen species (ROS) levels, which may be an effective alternative to existing stable embryo culture systems. In the same way, Clark et al. [186] designed microfluidic chips that mimic the function of the fallopian tube and created a pattern of sperm flowing through the oocyte, similar to the pattern in the fallopian tube. It was found that in vitro fertilization of oocytes in microchannels had a higher penetration rate of single sperm (p < 0.05) than traditional micro-drop fertilization, and the penetration rate was comparable to that of male pronucleus formation. A novel microfluidic device was able to observe sperm migration and select sperm with better motility and DNA integrity, providing a good basis for in vitro fertilization procedures to select sperm [187]. In order to study the correlation between early embryos and late fetal development, Marcia et al. [188] established a fallopian tube chip platform to better study the mechanism of gene reprogramming and the degree of difference between in vitro and in vivo embryos, thereby improving the quality of IVF fertilized eggs and gene integrity. A recent study has developed a microfluidic chip to create an adhesive surface and create a progesterone gradient to simulate the in vivo fallopian tube microenvironment [189], which revealed the entanglement process of sperm escape before fertilization in a microfluidic system (Fig. 4b). Taken together, these studies suggest that microfluidic bionic fallopian tubes hold significant potential for enhancing sperm selection strategies and improving physiological in vitro fertilization systems.

## Microfluidic chips for the uterus

The uterus is a crucial organ for the gestation of developing embryos or fetuses, boasting a histological composition of the luminal epithelium (LE), stroma, and myometrium. In mammalian species, the endometrium forms the uterine cavity, which undergoes dynamic changes in thickness and composition in response to the menstrual cycle [190]. Successful embryo implantation is contingent on the cessation of this cyclical uterine transition, whereby the uterus readies itself to accommodate the embryo. Following implantation, apoptosis occurs in the uterine LE cells at the attachment site, and the stromal cells differentiate into secretory decidual cells, providing a supportive matrix that reinforces the subsequent development of both the embryo and placenta. Hormonal disturbance or pathological conditions, such as uterine fibroids, adenomyoma, or carcinosarcoma, can adversely affect reproductive health in women [191]. Conventional clinical strategies such as surgery and chemotherapy remain the primary treatments for these uterine-related afflictions. However, animal models have been the mainstay for investigating the complexity of uterine-related diseases and their interaction with embryos during implantation. The limitations of such models have galvanized researchers to employ microfluidic chips and in vitro models to simulate the intrauterine environment, facilitating a more in-depth understanding of the processes of embryo implantation, placental development, and pregnancy complications.

### Endometrium

The endometrium represents a vital mucosal and epithelial layer lining the uterus, serving as the critical site of implantation for a fertilized embryo. Alterations in endometrial thickness, as well as diseases such as endometriosis, may adversely affect fertility outcomes or contribute to miscarriage. Endometrial receptivity describes the window of time during which the endometrium is poised to accept blastocyst attachment and invasion, generally occurring 6–10 days post-ovulation. Successful implantation will trigger a series of endometrial changes, culminating in the establishment of a functional placenta. Although considerable progress has been made through animal studies, significant interspecies divergence remains, warranting more human-relevant models. In vitro models capturing endometrial physiology are crucial yet challenging, necessitating improved approaches for obtaining and maintaining primary endometrial tissue cultures in multi-layer configurations.

#### Construction of microfluidic models of the endometrium

The endometrial microenvironment is a sophisticated system consisting of various endometrial cells, cytokines, metabolites, and blood vessels that play a crucial role in the establishment and maintenance of pregnancy. It has a considerable impact on endometrial receptivity, embryo implantation, immune tolerance, and other biological processes [192]. Furthermore, it is closely related to several gynecological diseases, exemplified by recurrent abortion and endometriosis [193]. Novel technologies and methodologies have emerged to improve or restore the endometrial microenvironment, such as exosome-hydrogel [194]. Nevertheless, the lack of a suitable in vitro model of human endometrial cells hampers the analysis of critical cell interactions, hindering the comprehensive comprehension of this fundamental aspect of reproductive tract function. Despite advances, there remains a growing need to simulate the endometrial microenvironment in vitro accurately. Doing so would enable a better understanding of the timing and spatial characteristics of cell communication between endometrial cell types, the interplay between endometrial stromal cells and adjacent vascular endothelial cells, and the influence of various hormone levels throughout the menstrual cycle. Ultimately, this would significantly advance women's reproductive health management.

The establishment of a microfluidic chip utilizing microfluidic properties to enable long-term co-culture of human endometrial stromal and endothelial cells in vitro has allowed for the simulation of ideal hormone time changes during the 28-day menstrual cycle and successful differentiation into functional decidual cells, as shown in Fig. 4c. This model of the endometrial perivascular stroma has been demonstrated to persist for up to four weeks and remain responsive to steroid hormones, enabling quantitative biochemical analysis [195]. Moreover, the investigation into the role of the vascular endothelium in early human endometrial perivascular decidualization has revealed that perfusion of the endothelium leads to increased sensitivity of interstitial fibroblasts to progesterone and augmentation of the decidualization response. Gnecco et al. [196] exploited microfluidic chips to study that perfusion of vascular endothelium enhances the sensitivity of interstitial fibroblasts to progesterone and enhances the decidualization reaction. It was also found that laminar shear stress regulates endometrial differentiation through endothelial prostaglandins, especially prostaglandin ovarian steroids estrogen and prostaglandin. An innovative micro-engineered vascularized endometrial chip has been designed utilizing three layers of epithelium, stroma, and blood vessels to reconstruct the physiologically relevant endometrial environment, and has been shown to effectively respond to hormone stimulation and angiogenic factors (Fig. 4d) [197]. This approach has facilitated the testing of the efficacy and safety of the emergency birth control drug levonorgestrel, and has enabled the establishment of an advanced in vitro model of embryo implantation, which provided an effective tool for drug screening and toxicity testing in the field of female reproduction. And also, the microfluidic verified that oxygenation-induced senescent AECs encapsulate HMGB1 into inflammatory cargoes in exosomes and spread to maternal uterine cells, increasing inflammation and acting as a signal for the fetus to initiate delivery, shown in Fig. 4e [198]. Collectively, the microfluidic chip can simulate the microenvironment of the endometrium and the changes related to the menstrual cycle, while promising to aid in the deeper elucidation of the complex mechanisms underlying endometrial health and disease.

#### Co-culture microfluidic model of endometrium and embryo

The co-culture of endometrial cells and embryos offers a promising way to simulate the intricate milieu of implantation in vivo, thereby enhancing the success rate of embryo development and pregnancy. This strategy can promote embryo differentiation and implantation by stimulating the secretion of growth factors and cytokines, such as interleukin-1, interleukin-6, and transforming growth factor-β by endometrial cells [199]. In addition, it increases the adhesion of embryos within the uterine cavity by creating a dendritic structure that firmly wraps the embryo and prevents its displacement [200]. There are two common ways to co-culture embryos and endometrial cells, while direct contact involves placing embryos and endometrial cells together in the same dish to mimic the in vivo implantation process, the indirect method uses a semi-permeable membrane or small chamber to enable cytokine exchange between the two cell types [201]. Both methods have limitations such as difficulty distinguishing factors from different cell sources, controlling the ratio between cells, susceptibility to interference from microorganisms and pollutants, and inability to observe morphological changes [202]. To overcome these shortcomings, researchers have developed several advanced co-culture methods that combine the benefits of both direct and indirect contact approaches. For example, the conditional medium co-culture leverages the conditioned medium of endometrial cells to simulate the internal environment and facilitates the regulation of communication between cells [203, 204]. Other methods, such as the climbing plate co-culture [205], have also shown promise in mimicking the implantation environment and controlling cell communication. However, while these approaches offer advantages, they still have limitations such as the need for carrier treatments and failure to provide real-time cytokine changes.

The integration of microfluidic technology with co-culture technology provides a promising approach to establishing a biomimetic microenvironment for embryonic growth and development. By designing a unique microchannel structure, the microfluidic chip enables the co-culture of embryos and endometrial cells, resulting in improved embryonic development rates compared with traditional culture methods. Recent research has demonstrated that this approach can enhance blastocyst development rates by up to 16.1% when embryos are co-cultured with stromal cells on the chip [206]. The combination of microfluidic technology and co-culture technology can not only improve the quality of embryos developed in vitro, but can also automatically capture a single embryo. The use of dynamic medium perfusion in microfluidic systems closely simulates the endometrial microenvironment, enabling the efficient capture of single mature embryos for transplantation [207]. After 2–3 days of embryo culture in the chip, a single mature embryo can be taken out at one time for implantation regardless of the location of the culture chamber. However, further research is needed to fully explore and optimize the potential of this microfluidic co-culture model.

### Placenta

As a semi-permeable barrier comprised of embryonic trophoblast cells and maternal endometrial chorion, the placenta governs metabolic coordination between mother and fetus [208]. The placenta can sense the nutritional needs of the fetus, coordinate the maternal nutritional supply, and regulate its nutritional transport capacity [209]. The placenta is also an important endocrine organ that secretes more than 100 peptide hormones and steroid hormones to maintain normal pregnancy [210]. To understand the intricacies of this crucial structure, as well as the impact of various exogenous factors, researchers have developed in vitro models of the placenta, which can simulate the physiological environment, establish placental circulation, and evaluate the integrity and functionality of villi [211]. By investigating the workings of such models, researchers may uncover new mechanisms of disease, treatment strategies, and preventative measures. At the same time, an effective method is needed to simulate the placental interface between maternal and fetal blood in vitro to reflect the exchange of nutrients between mother and fetus [212, 213]. Notably, the microfluidic in vitro placental model is the most advantageous research model for drug placental transport and trophoblast cell simulation, which can provide new ideas for safe medication during pregnancy.

**Fig. 5:**
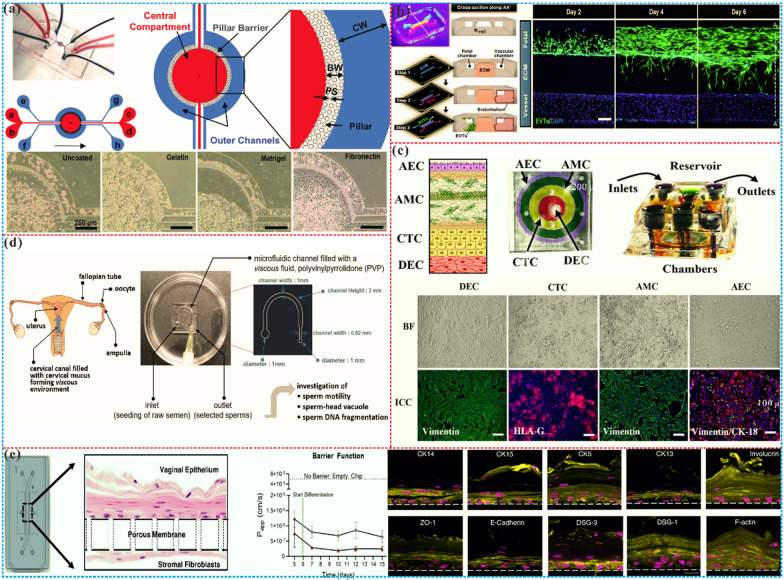
Microfluidic chips for placenta and cervix. **a** A microfluidic chip was utilized to simulate placental trophoblast cells invading the maternal uterus as the maternal–fetal interface [217]. Copyright 2021 Royal Society of Chemistry. **b** The critical process of embryo adhesion to the endometrium and subsequent invasion of maternal tissue implantation and the placenta was reproduced in vitro using a microfluidic chip [218]. Copyright 2021 Springer Nature. **c** A human placenta-maternal interface organ chip was constructed using a microfluidic chip to test the pharmacokinetics and efficacy of the drug [222]. Copyright 2022 Royal Society of Chemistry. **d** Microfluidic sperm sorting chip to simulate human cervical canal biophysical environment simulation system [227]. Copyright 2021 MDPI. **e** The vaginal organ chip model was used to better understand the interaction between vaginal microbiome and host tissue, and to evaluate the safety and efficacy of live biotherapy products [237]. Copyright 2022 Springer Nature

To investigate the impact of placental drugs on maternal and child health, prior studies have employed various methods, including animal experiments, cell culture, and population observation [214]. However, due to significant differences in physiological structures between animals and humans, it is challenging to identify a suitable model that can fully mimic the complex processes occurring in the human placenta [215]. Consequently, research findings have often been inconsistent or inaccurate. Recent developments in microfluidic devices have shown promise in providing more accurate, efficient, and cost-effective platforms to study placental interactions [216]. Pu et al. [217] proposed a placenta platform on a chip, which replicates pivotal elements of the placental microenvironment, including endothelial and trophoblast cells, layered with extracellular matrix and integrated with dynamic media flow, while allowing real-time monitoring, imaging, and evaluation of trophoblast invasion and inter-cellular interactions (Fig. 5a). And the microfluidic barrier model combined with natural blood flow profiles can be used to simulate the placental microenvironment and study different drug, clinical and biological scenarios [218]. A microfluidic chip implantation technology has been exhibited in Fig. 5b, which can reconstruct the three-dimensional structure of the maternal–fetal interface to simulate the invasion of specialized fetal extravillous trophoblast cells into the maternal uterus. Using human primary cells isolated from clinical specimens, it demonstrated the in vivo directional migration of extravillous trophoblast cells to micro-engineered maternal blood vessels and their interaction with endothelial cells necessary for vascular remodeling [219]. Another microfluidic barrier model has been developed to combine natural blood flow profiles, allowing the placental microenvironment to be replicated and facilitating the study of various drug and biological scenarios [220]. The chip was designed to carry two cell lines, representing the mother and the fetus, respectively. A porous membrane is placed between the two channels as a barrier between the two blood flows. This membrane acts as an extracellular matrix to provide support for surrounding cells. Moreover, the use of drugs during pregnancy is of particular concern, not only for the health of pregnant women, but also for the effects of drugs on offspring, and the effects of caffeine on the fetus when passing through the placental barrier were studied [221]. Advanced research in Fig. 5c also utilized microfluidic chips to represent the structure, function, and response of the human placenta-maternal interface [222]. Among them, the placental chip models consist of placental villi, chorionic villi and amniotic membrane, while the fetal membrane-decidual interface chip model consists of decidua, endometrium and myometrium. The results proved that these chip models can accurately simulate the structure and function of the human placental-maternal interface and can transport and metabolize drugs. In addition, the study demonstrated that the use of these chip models can determine the safety and efficacy of drugs. These advancements will contribute to a more robust understanding of the interactions between the mother, fetus, and drugs and ultimately improve maternal and child health outcomes.

### Cervix

The cervix is situated in the inferior part of the uterine region. The primary function of this structure is to safeguard against external infections, while concurrently maintaining the vaginal pH balance and facilitating sperm motility and survival. And the cervical mucus, which is secreted by the cervix, plays a pivotal role in the selective process of identifying fit and healthy spermatozoa during the process of natural fertilization [223]. Although the dynamics of sperm in viscous media formed from hyaluronic acid and methylcellulose have been studied, the mechanisms underlying these phenomena remain unclear [224]. Also observed was the effect of changes in the viscosity of these media with microfluidic on sperm motility [225]. But a reliable method is still needed to analyze the direct relationship between other important characteristics, such as sperm head morphology and DNA integrity [226]. Park et al. [227] proposed a microfluidic chip utilizing a polyvinylpyrrolidone solution as a viscous medium, which replicates the cervical mucus environment (Fig. 5d). The sperm identified by the cervical chip exhibited excellent motility, normal morphology, and high DNA integrity. This innovative approach sheds light on the fundamental mechanisms behind natural selection and sperm navigation during fertilization within the female reproductive system.

## Microfluidic chips for the vagina

The vagina is an important part of the female reproductive system and is a conduit connecting the uterus and external reproductive organs. The significance of vaginal health for women's reproductive systems cannot be overstated. The occurrence of various vaginal issues, such as vaginitis, vaginal dryness, and vaginal relaxation, can have a notable impact on women's reproductive health [228, 229]. Moreover, these conditions can potentially spark further reproductive diseases, such as cervicitis, endometritis, etc. [230, 231]. To ensure the effective prevention of such problems, a comprehensive understanding of the vaginal microenvironment is essential. The microbial community of *Lactobacillus*, anaerobic bacteria, and Gram-positive bacteria mainly constitutes the vaginal microenvironment, which forms an ecological balance and helps prevent infections [232–234]. Maintaining the balance of the vaginal microenvironment requires not only microbial equilibrium but also proper vaginal secretion, which has crucial antibacterial and nutritional components [235]. An imbalance in vaginal pH value, microbial community disorder, or other factors may result in vaginal infections and other diseases. Therefore, it is crucial to maintain a balanced vaginal microenvironment for optimal vaginal health that can prevent the occurrence of diseases.

The microfluidic vaginal chip is an innovative technology that has shown promise in the diagnosis and treatment of vaginal health issues. This tool utilizes the high sensitivity and throughput of microfluidic chips to conduct biological reactions in small spaces, resulting in quantitative analysis of cells and molecules [236]. Notably, the microfluidic vaginal chip has potential applications in the diagnosis and treatment of gynecological diseases, including bacterial and viral infections, as well as abnormal leucorrhea. To achieve this, researchers implanted human vaginal epithelial cells into the top channel of a polymer chip, separated from human uterine fibroblasts on the other side of the permeable membrane. A novel work displayed in Fig. 5e created a 3D structure that accurately replicated the human vaginal wall [237]. The chip produced multiple layers of differentiated cells that matched those found in human vaginal tissue after five days. It also allows scientists to introduce different strains of bacteria to study their impact on organ health, replicating the microenvironment of human vaginal tissue in vitro [238]. These capabilities make the microfluidic vaginal chip an essential tool for researchers seeking to understand the microenvironment of human vaginal tissue and develop new treatments to mitigate reproductive tract infections, prenatal complications, and infant mortality. Nevertheless, due to the higher technical thresholds and stricter quality control requirements, the cost of microfluidic vaginal chips is also higher. Thus, even though microfluidic vaginal chips are still at the exploratory stage in practical clinical application, they represent a significant advancement in women's health. Consequently, further research is required to confirm their reliability and accuracy.

## Microfluidic chips for gynecological diseases

### Microfluidic chip uterine disease model

Uterine diseases encompass a range of pathologies that affect the endometrium, myometrium, and cervix, including both benign and malignant tumors, infections, endocrine disorders, and adhesions. Uterine fibroids, endometriosis, endometrial cancer, and cervical cancer are among the most commonly encountered uterine diseases [239]. Despite extensive clinical management options, such as surgical interventions, radiotherapy, and chemotherapy, the underlying biological mechanisms governing these diseases remain incompletely understood. Therefore, there is an urgent need to establish human uterine disease models to unlock new insights into their underlying pathogenesis and devise innovative treatment strategies. Organoids, which allow for precise gene manipulation and can serve as personalized disease avatars, provide an attractive avenue toward this goal [240]. Inherent challenges in organoid scalability, repeatability, and consistency limit their applications in drug screening and high-throughput testing. Overcoming these limitations through microfluidic technology could pave the way for in vitro studies that simulate the pathogenesis of uterine diseases and screen for candidate drugs.

#### Endometriosis

Endometriosis is a complex and debilitating disease characterized by the growth of endometrial tissue outside the uterus. The underlying pathophysiology of this disease remains elusive, underscoring the need for the development of in vitro models to better understand the mechanisms of endometriosis [22]. In recent years, microfluidic technology has emerged as a powerful tool for creating physiologically relevant models of endometrial diseases. These systems enable the precise manipulation of the cellular microenvironment, allowing for the co-culture of various cell types, such as endometrial stromal cells, immune cells, and fibromuscular cells, to more accurately reflect the complexities of the diseased tissue. Microfluidic fabrication of in vitro endometrial models can elucidate crucial cellular and molecular determinants in pathologies and screen pharmacological agents, facilitating the acquisition of research materials, and reducing ethical dilemmas and expenses [241]. By recapitulating the interactions between endometrial stromal cells and peritoneal mesothelial cells in patients with endometriosis, these models have shed light on the role of peritoneal physiology in the development of endometriosis [242], which found that endometriosis can invade endometrial stromal cell and separate peritoneal mesothelial cells, indicating that peritoneal physiology has an important impact on the development of endometriosis. Subsequent models also added stromal cells, immune cells and fibromuscular cells to better simulate the microenvironment of endometriosis lesions [28, 195]. These micro-physiological systems have also allowed for the measurement of matrix metalloproteinase activity and mechanical properties of endometrial cells in patients with endometriosis, providing insights into the pathophysiology of this disease [243, 244]. Additionally, several studies have incorporated the peristaltic motion of the uterine smooth muscle layer, which is altered in patients with endometriosis, to more accurately mimic the disease microenvironment [245–247]. This is important for the modeling of endometriosis because the patient’s uterine contraction frequency is times higher than normal women [248]. The micro-physiological system can also achieve long-term co-culture of endometrial cells with other related cells (such as immune cells, mesothelial cells and perivascular cells). The ability of these microfluidic models to accurately reproduce cell-to-cell and cell-to-extracellular matrix interactions, as well as tissue-specific structures and physicochemical microenvironments, provides unprecedented opportunities for studying the communication between ectopic and adjacent cells and for analyzing the specific paracrine cross-talk between cells. Entirely, microfluidic organ models have the potential to greatly enhance our understanding of endometriosis, ultimately leading to the development of more effective treatments and improved patient outcomes.

#### Uterine fibroid

Uterine fibroids are non-cancerous tumors that arise from the proliferation of smooth muscle cells within the uterus, and can lead to a variety of debilitating symptoms. Their pathogenesis involves both intercellular and extracellular mechanisms, and is associated not only with the uterus, but also with systemic vascular disorders, including hypertension, preeclampsia, and atherosclerosis [249]. Minimally invasive surgical procedures currently represent the mainstay of treatment for uterine fibroids; however, in vitro models are critical for investigating their underlying mechanisms and developing novel therapeutic approaches. A recent study by Banerjee et al. [250] utilized normal and patient-derived muscle stem cells to create 3D organoid structures that faithfully recapitulated the tissue architecture, differentiation capacity, and hormone responsiveness characteristic of uterine fibroids. Despite this promising progress, there remains an unmet need for improved in vitro models which can capture the complex extracellular and microenvironmental factors involved in uterine fibroid pathogenesis. Microfluidic devices hold tremendous potential for fulfilling this requirement by enabling the simulation of various physiologic and pathologic processes in a precise, controlled, and dynamic manner. Future research focused on developing microfluidic-based platforms for uterine fibroid modeling may provide novel insights into their underlying biology and identify new therapeutic strategies for addressing this debilitating disease.

### Preeclampsia

Preeclampsia, fetal growth restriction, and stillbirth are grave pregnancy complications that pose a significant threat to maternal and fetal health. These complications stem from placental dysfunction caused by the inadequate invasion and transformation of uterine arteries by placental extravillous trophoblast cells [251]. And the molecular mechanisms responsible for controlling trophoblast invasion and regulating spiral arteries are not well understood. The traditional approach to study cell invasion, the transwell assay, is not suitable for primary trophoblasts due to the challenge of cell number requirements and the issue of non-trophoblast contamination [252]. Abbas et al. [253] exploited a novel in vitro microfluidic model that retained the physiological correlation of trophoblast cells in three-dimensional invasion and it is introduced to study the migration behavior of human primary trophoblast cells. Moreover, this microfluidic chip model highlights the importance of endothelial cells in trophoblast-mediated migration, which can effectively reconstruct the endothelial vascular network to simulate placental development and vascular dysfunction in preeclampsia [254]. As a gonadal protein, follistatin (FST) has been demonstrated to enhance the viability, proliferation, and wound healing of primary cultured embryonic trophoblast cells [255]. This process has been further corroborated in the microfluidic device, revealing that FST acts as a chemokine to promote JNK signaling pathway-driven trophoblast cell migration and invasion, ensuring trophoblast cell function, and advancing placental growth [256]. The employ of microfluidic devices presents an opportunity to examine the multifaceted physical and chemical impacts on trophoblast behavior during placental formation, offering new insights into the intricate mechanisms at work.

### Gynecological cancers

Female malignancies encompass a multitude of cancer types, the most concerned including ovarian, endometrial and cervical. The incidence and mortality rates of these cancers are influenced by age, race, genetic and environmental factors. For instance, cervical cancer predominantly arises from human papillomavirus (HPV) infection, while endometrial cancer may be connected to genetic conditions such as Lynch syndrome, and ovarian cancer may be linked to BRCA1/2 gene mutations [257]. Additional correlative factors include fertility history and obesity. Each type of cancer displays distinctive prevention, symptoms, diagnosis, and treatment modalities. Currently, clinical surgery and chemotherapy serve as the primary forms of diagnosis and treatment for gynecological cancer. However, there remains a lack of thorough comprehension and preventative strategies against gynecological malignancies. Novel in vitro models that better mimic the pathogenesis of these cancers and provide insight into effective treatment plans are thus necessary. The tumor microenvironment entails a complex network comprised of multiple cell types and molecules, rendering it challenging to accurately reproduce in vitro. 3D culture models offer a means to simulate the physiological microenvironment more accurately via micro-assembly structures and alteration of pertinent parameters. It can promote cell differentiation and tissue growth by using micro-assembly structures and regulating complex environmental parameters. Hydrogels, spheres, tissue scaffolds, and microfluidic chips represent various 3D culture systems, each harboring their own respective strengths and weaknesses [258]. For instance, hydrogels can effectively mimic tissue elasticity and biological activity, but can be lacking in stability and repeatability. Spheres may be quickly and easily prepared, but are limited by their lack of spatial structure and nutrient exchange. Tissue scaffolds provide robust mechanical support and high permeability, but possess drawbacks such as insufficient biocompatibility and degradability. Microfluidic chips, however, hold potential by allowing accurate control over fluid dynamics and chemical gradients, serving as a valuable engineering methodology for early diagnosis of gynecological cancer and as a platform for constructing in vitro tumor models for drug-mediated treatment strategies [259].

#### Ovarian cancer

**Fig. 6:**
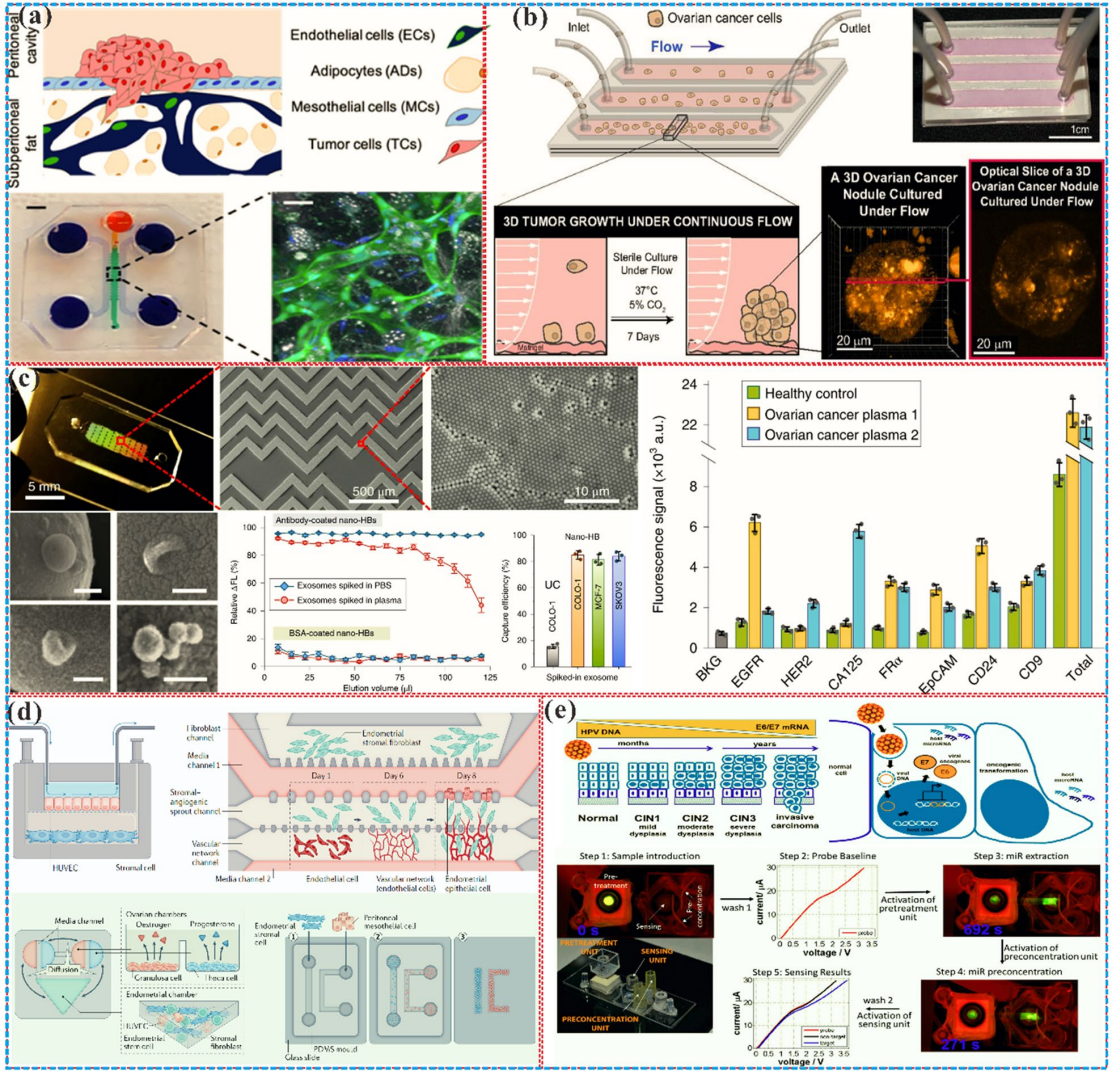
Microfluidic chips for gynecological cancers. **a** The effects of stromal cells on the attachment and growth of tumor cells in early and late metastasis models were studied by using human peritoneal omentum and ovarian tumor microenvironment vascularization microfluidic model in vitro [264]. Copyright 2022 Elsevier. **b** Using 3D ovarian cancer nodules to study the role of fluid force as a biological regulator in metastatic cancer [265]. Copyright 2013 PNAS. **c** Microfluidic chip can detect lower levels of ovarian tumor-associated exosomes in plasma [267]. Copyright 2019 Springer Nature. **d** Construction of microfluidic models for uterine diseases such as endometriosis, adenomyosis, and endometrial cancer [22]. Copyright 2022 Springer Nature. **e** A microfluidic nucleic acid-based biosensor to evaluate circulating host microRNAs in response to oncogenic human papillomavirus infection and related detection of cervical cancer [284]. Copyright 2016 MDPI

Ovarian cancer remains one of the most prevalent malignancies affecting female reproductive organs, with a multifactorial etiology incorporating age, fertility, blood type, mental factors and environment. This malignancy often evades timely detection due to non-specific early symptoms, culminating in late diagnosis and poor outcomes. Treatment modality selection for ovarian cancer hinges on the stage, grade, genetic status, and tumor type, with established interventions ranging from targeted therapy to neoadjuvant chemotherapy, surgical debulking, immune checkpoint inhibitors, and hormone therapy [260]. Despite advances, the treatment of recalcitrant ovarian tumors remains challenging. In recent years, the advent of microfluidic technology has provided a promising avenue for enhancing ovarian cancer management, which offers micron-scale channels and chambers, delivering superior precision in analyzing ovarian tumors via chemical reaction technology [261]. This approach has stimulated the development of novel detection platforms for ovarian cancer biomarkers, offering renewed hope for patients with complex tumors refractory to conventional protocols. A typical method for constructing an in vitro model of ovarian cancer is to use matrix-coated culture dishes and media as spherical or tumor explants to promote cancer cell proliferation [262]. Microfluidic spherical culture presents a promising alternative with micro-cancer dimensions, minimizing the cell requirement during initiation, and ensuring stability, and consistency in drug response [263]. Furthermore, microfluidic chips authentically replicate the ovarian tumor microenvironment, mimicking oxygen gradients, nutrients, drug responses, growth, migration, invasion, and apoptosis, as shown in Fig. 6a and b [264, 265]. Overall, microfluidic technology represents an attractive tool for understanding, characterizing, and treating ovarian cancer.

The advent of microfluidic-based biosensor systems represents a promising avenue for the quantitative detection of molecular biomarkers associated with a range of debilitating diseases. In particular, the development of advanced microfluidic platforms for the detection of ovarian cancer stands to revolutionize clinical diagnosis and patient monitoring [266]. A microfluidic chip designed with a self-assembled three-dimensional herringbone nanopattern can detect lower levels of ovarian tumor-associated exosomes in plasma (10 μl^−1^, or about 200 vesicles per 20 μl of sample added), as shown in Fig. 6c. This nanopatterning device for nanoimaging should facilitate the use of liquid biopsy in ovarian cancer diagnosis [267]. And also, the microfluidic chip can achieve multiple exosome detection, and improve sensitivity and specificity. Zhao et al. [268] showcased the efficacy of a microfluidic nanoprobe to demonstrate that circulating exosome CD24, epithelial cell adhesion molecule (EpCAM) and folate receptor α (FRα) markers can be quantitatively detected using only 2 μl plasma to detect ovarian cancer. Similarly, the use of microfluidic devices to detect exosome proteins (pSTAT3, HGF and IL6) in serum exosome samples will facilitate the development of new targeted therapies based on HGSOC-specific signaling pathways [269]. Using microfluidic chip technology to screen the specific binding of oligopeptides to ovarian cancer cells is also an effective strategy for early diagnosis of ovarian cancer [270]. Additionally, high expression of the FXYD2 gene is closely related to ovarian clear cell carcinoma, which is a highly lethal ovarian cancer subtype, and the use microfluidic chip can achieve rapid, sensitive, specific and quantitative detection of the FXYD2 gene [271]. Such systems have shown great promise in enabling the simulation and mechanism analysis of ovarian cancer microenvironments and facilitating early detection and improved management of this devastating disease.

#### Endometrial cancer

Endometrial cancer is a malignancy arising in the endometrium, typically presents with abnormal vaginal bleeding and may also cause pelvic discomfort or abdominal masses. Diagnosis generally involves biopsy or cystoscopy with preoperative staging evaluation and molecular marker detection [272]. Primary treatment involves surgical resection, including total hysterectomy, bilateral adnexectomy, and pelvic lymph node dissection, while adjuvant therapies such as radiotherapy or chemotherapy may be considered for high-risk or relapsed patients [273]. In general, patients with early and low differentiation have a better prognosis, while patients with advanced and highly differentiated have a worse prognosis. 3D cell culture of endometrial cancer cells compared with 2D monolayer cell culture, cells in 3D culture showed better potential for tumor microenvironment simulation [274]. Mostly, in vitro studies on endometrial cancer are based on organoid models. By introducing fibroblasts isolated from endometrial cancer lesions to optimize the current endometrial cancer organoids, the disease-related characteristics and cancer-related mutations were studied [275]. Organoids faithfully reproduce the genotype of the disease, which will be valuable for exploring the proliferative phenotype and the molecular mechanism of cancer development [276]. However, their limitations in terms of time-consuming and costly culture pose a challenge to early diagnosis and real-time analysis. Microfluidic chips have the potential for early diagnosis and in vitro research analysis, yet their application in endometrial cancer remains largely unexplored. Appropriate microfluidic model systems that closely mimic the structure and function of endometrial-related diseases are necessary (Fig. 6d) [22].

#### Cervical cancer

Cervical cancer, a prevalent malignant tumor afflicting the female cohort, is predominantly induced by the HPV. Disturbingly, the incidence and mortality rate of cervical cancer is higher than other gynecologic malignancies [277]. The treatment plan for cervical cancer depends upon the diagnosis stage and may comprise radical hysterectomy, chemoradiation therapy, or a combination of both [278]. Conservative surgery to preserve fertility has become the standard treatment for patients with low-risk, early-stage lesions. For patients with locally advanced lesions, advancements in radiotherapy techniques such as intensity-modulated radiation therapy have significantly lowered the toxicity rate [279]. Despite current treatment regimens, patients with metastatic or recurrent lesions experience a bleak prognosis. Nevertheless, the incorporation of the anti-vascular endothelial growth factor drug bevacizumab has lengthened overall survival by over twelve months [280]. Novel immunotherapeutic modalities, emulating that of other solid tumors, have shown promising preliminary results [281]. Currently, HPV vaccination is an effective and conventional preventive measure against cervical cancer [282].

HPV testing is a pivotal approach in cervical cancer screening that is commonly conducted via HPV DNA or microRNAs testing, Pap cytology, colposcopy and biopsy, which are considered the gold standard in this field [283]. Microfluidic technology can facilitate rapid, sensitive and specific detection of HPV DNA, thereby offering convenience in the early diagnosis and prevention of cervical cancer [284, 285], as shown in Fig. 6e. Immunotherapy has emerged as a promising treatment strategy that leverages the body's immune system to combat tumor cells, and has shown remarkable outcomes in various cancer types [286]. Microfluidic technology is being increasingly employed to prepare personalized tumor immune vaccines or isolate and amplify specific immune cells, thereby enhancing the efficacy and safety of immunotherapy. Inan et al. [287] have reported on a customized immunoassay platform that interfaces with a microfluidic filter to detect and quantify anti-HPV16 E7 antibodies in whole blood, serving as a non-invasive adjunct technique for diagnosing HPV-related malignancies. The chip-based antibody detection system has been found to effectively identify and capture cervical cancer cells in heterogeneous cell populations [288]. By incorporating antibody channels onto microfluidic chips and allowing for physiologic conditions to guide cervical cancer cell capture, the potential utility of microfluidic chips in cervical cancer diagnosis and immunotherapy is elucidated. The amalgamation of microfluidics with nanomaterials has yielded innovative approaches to detecting cancer-related biomarkers [289]. Gu et al. [290] have fabricated a microfluidic detection platform based on an Au@SiO_2_ array with a highly surface-enhanced Raman scattering (SERS) active substrate that permits the simultaneous detection of squamous cell carcinoma antigen and carcinoembryonic antigen in clinical serum samples, underscoring the potential of microfluidic chips for clinical cervical cancer diagnosis. Drug intervention, such as tannin from Spatholobi Caulis (TTS), has also emerged as a significant treatment modality for cervical cancer. The efficacy and mechanism of TTS on relevant proteins were analyzed via a molecular docking microfluidic platform, and considering statistical analysis for the 3D microfluidic in vitro model, and bioinformatics integration [291]. These studies provide a foundation for the application of this drug in cervical cancer treatment.

## Human-assisted reproductive technology

**Fig. 7:**
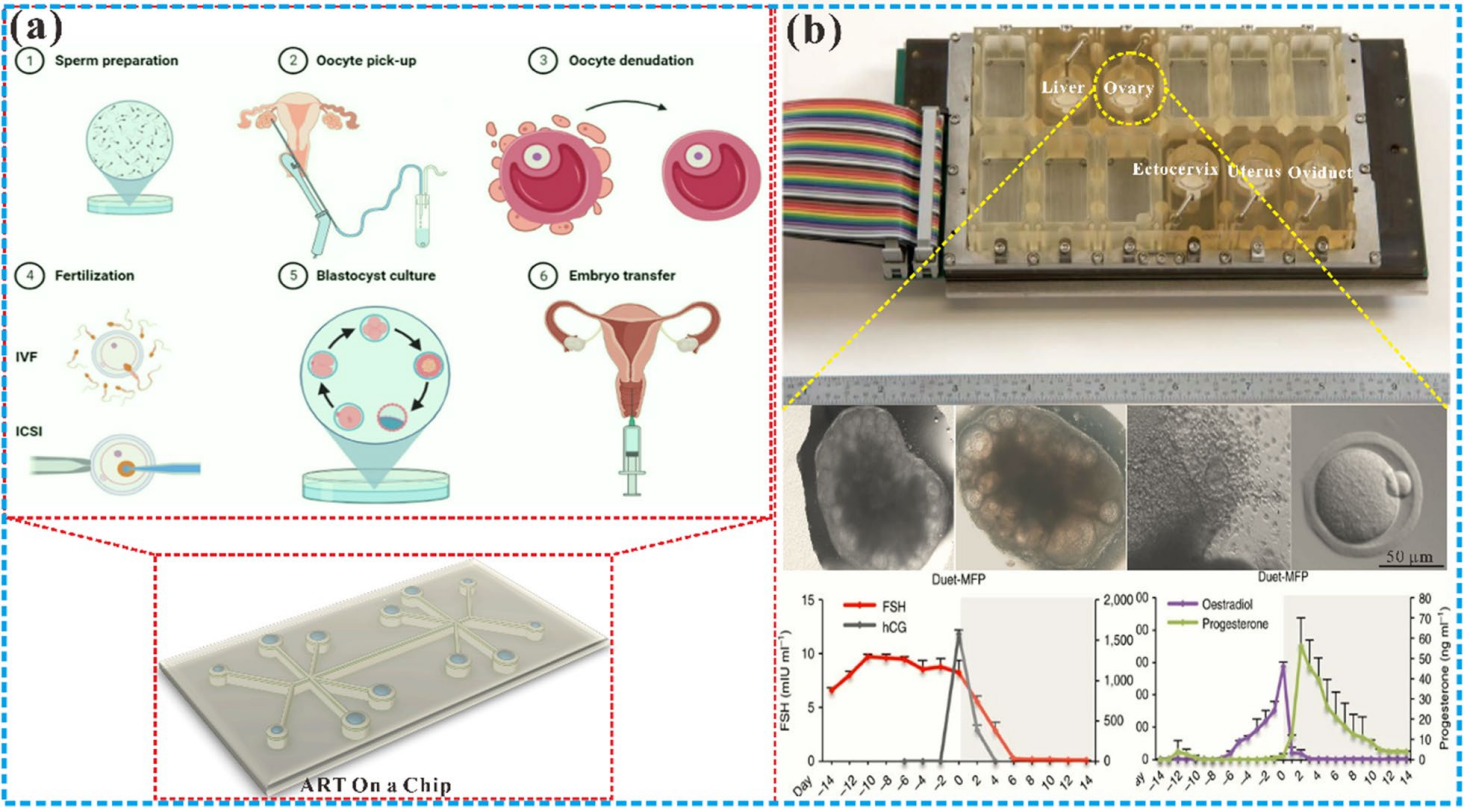
**a** ART on the microfluidic chip. Reprinted with permission from Ref [23]. Copyright 2023 Elsevier. **b** Integrated genital tract tissues and peripheral organs into a multi-unit integrated microfluidic platform [123]. Copyright 2017 Springer Nature

The issue of fertility disorder, caused by male sperm deficiency or low sterility in the female reproductive system, is a major concern. ART has emerged as an effective solution to address these issues through the manipulation of eggs, sperm, fertilized eggs, and embryos using medical techniques, including artificial insemination, in vitro fertilization-embryo transfer, intracytoplasmic sperm injection technology, and preimplantation genetic testing [292]. In the last decade, ART has made substantial progress, in providing solutions to infertility. Nonetheless, ART raises several ethical dilemmas, such as the lack of uniform medical standards, quality control issues, and the need for improved supervision, publicity, and education [293]. Moreover, inadequate control over the quality of sperm could prevent the fetus from being guaranteed, the microfluidic technology has gained attention in the field of ART due to its potential to innovate traditional IVF procedures [294, 295]. Microfluidic technology can be combined with a variety of ART procedures, such as embryo acquisition, sperm screening, analysis, classification, operation, culture and monitoring [296]. The schematic diagram of ART on a microfluidic chip is shown in Fig. 7a. These advancements overcome ethical issues and improve the efficiency of sperm selection and in vitro fertilization. While there have been many reviews on the advantages of microfluidic ART [23, 297–301], the focus of this review is to analyze the current status of microfluidic technology and its future potential. With the continuous development of microfluidic technology, the integration, automation, intelligence and standardization of assisted reproductive technology will be the direction of future development. Improving the multi-functionality of microfluidic chips will further liberate manpower and make the operation and selection of gametes and embryos more accurate and efficient. This will provide a superior solution platform for human reproductive disorders.

## All-in-one microfluidic chips of FRS

The integration of microfluidic technology into the field of female reproductive system research presents numerous potential benefits. This innovative approach allows for precise co-culture of cells, real-time detection of cell-related factors, and accurate completion of in vitro culture of reproductive system cells with increased speed and efficacy. By incorporating perfusion dynamic culture chambers, screening channels, micro-nano surgical operation platforms, and biosensors onto a single chip, traditional methods can be streamlined and made more efficient. Additionally, the creation of a microfluidic device that simplifies the treatment and operation of oocytes and allows for automatic tracking of embryo development has significant implications for in vitro fertilization practice. The microfluidic device can simplify the treatment and operation of oocytes, allow rapid and convenient replacement of media, and can automatically track the development of any single embryo [154], thus bringing some advantages to in vitro fertilization practice. Moreover, by utilizing integrated microfluidic chips, researchers can study the intricate and interconnected mechanisms that regulate female reproductive systems in greater detail to develop informed solutions to female health issues. Xiao et al. [123] integrated genital tract tissues and peripheral organs into a multi-unit integrated microfluidic platform called EVATAR to simulate the endocrine circuit between the female genital tract and the modules of ovaries, fallopian tubes, uterus, cervix and liver in vivo, and continuously circulated among all tissues (Fig. 7b). The above research points out the direction for the integrated microfluidic chip of female reproductive system, which can simulate the related mechanisms and microenvironment in the FRS more deeply and systematically, and provides a good strategy for promoting the solution of female health problems.

## Summary

The deployment of microfluidic platforms promises to revolutionize the landscape of women's healthcare, bolstering diagnostic precision and expediting clinical workflows. This innovative approach harbors the potential to tailor therapeutic strategies to individual patient profiles, thereby to optimize outcomes and curtail healthcare expenditures. Such advancements stand poised to make a pivotal impact on enhancing reproductive health and elevating the standard of women's well-being universally. This review delineates the pivotal role of microfluidic chip technology in elucidating and managing female reproductive health. Microfluidic devices boast an unparalleled precision in emulating the intricate cellular microenvironments, thereby facilitating a spectrum of experimental paradigms that transcend the confines of traditional modelling approaches to intricately replicate ovarian cellular processes. The synthetic microfluidic fallopian tubes herald a new era for augmenting sperm selection methodologies and refining physiological in IVF protocols. Utilization of microfluidic chips for in vitro modelling simulates the uterine milieu, offering profound insights into embryo implantation, placental morphogenesis, and gestational complications, as well as pathologies such as endometriosis. Notably, the in vitro microfluidic placental model emerges as the quintessential research paradigm for investigating drug translocation across the placenta and trophoblast cell dynamics, thus inspiring novel paradigms for pharmacological safety during pregnancy. The microfluidic representation of the cervical milieu aims to unveil the rudimentary mechanisms of gamete selection and navigation within the fertilization pathway, enhancing the selection of viable spermatozoa. The deployment of microfluidic platforms in gynecological pathology is of paramount importance, given the incomplete understanding of the biological underpinnings associated with conventional treatment modalities. There is a pressing imperative to establish gynecological disease models, including uterine and oncological representations, to unearth their potential etiologies and spearhead cutting-edge therapeutic strategies. The scalability, reproducibility, and consistency inherent in microfluidic technology amplify its utility in drug screening and high-throughput assays, paving the way for in vitro simulation of gynecological disease pathogenesis and candidate pharmacological screenings. ART and IVF on microfluidic substrates not only circumvent ethical conundrums but also enhance the efficiency of gamete selection and fertilization processes. An integrated microfluidic device can expedite and refine the in vitro culture of reproductive cells, surpassing traditional methods in speed and efficacy. The convergence of dynamic perfusion culture chambers, selective conduits, micro-nano surgical interfaces, and biosensors into a singular chip streamline and elevates both the screening and therapeutic landscapes. Despite the inherent challenges, the relentless progression of microfluidic methodologies portends a transformative impact on women's healthcare by ameliorating diagnostic and therapeutic modalities.

## Future directions and challenges

Currently, most microfluidic chips can only achieve low throughput. Microfluidic chip platforms with medium and high throughput can simulate more complex tissue-tissue or organ-organ interactions, which will be more useful for pre-clinical single or double-organ toxicity and effectiveness studies. Multi-organ microfluidic systems have the promise to reduce the necessity for animal research. Though intricacies entail their development, ongoing scientific and technological advancements suggest that there is considerable potential for improvement in multi-organ integrated microfluidic platforms. By fusing biosensors with these platforms, high-sensitivity, biological signal detection microfluidic chips can be created in real time for individualized disease diagnosis. Microfluidic chips are adept at analyzing and processing voluminous data; they offer critical support in predicting, diagnosing, and treating diseases, while concurrently simulating the function of human organs, exploring disease mechanisms, exploring pharmaceutical toxicity and effectiveness, and developing new drug discoveries. With the popularity of an artificial intelligence (AI) named ChatGPT [302], AI in conjunction with microfluidic chips assists medical research in further comprehending intricate physiological and disease mechanisms within the human body. By simulating the growth and diffusion process of cancer cells through microfluidic chips and analyzing this data with AI algorithms, personalized treatment plans can be predicted. Additionally, artificial intelligence tools, like deep learning neural networks, can estimate drug effectiveness and toxicity based on microfluidic chip simulation experimental data, thus guiding new drug inventions. By applying microfluidic chips with AI algorithms, not only can female reproductive health be better researched, but the accuracy and efficiency of disease prediction, diagnosis, and treatment advancement across the medical field, contributing significantly to human health.

The cutting-edge organ chip model offered the opportunity to replicate the tissue and organ-level functionalities of living systems in vitro. This approach has been successfully applied to the female reproductive system, facilitating the precise diagnosis and treatment of individuals while detecting diseases in the female reproductive system with high accuracy and minimum sample size in future applications. Specifically, fetal DNA can now be identified in the serum of pregnant women, revealing the susceptibility of the offspring to viral infections and allowing for further study of their impact. And that is a direction that has not yet been reported. Moreover, microfluidic chips have been used to study the hormone regulation mechanism of the reproductive system. The complex process of interaction between individual cells, tissues and organs in the reproductive system and the nervous system can be visualized to better study the hormone regulation mechanism in the female reproductive system. For instance, integrated chips have been developed to model various organs such as ovaries, uterus, and breasts in microfluidics to simulate the interaction between organs at different hormone levels, enabling a better understanding of the hormone regulation mechanism in the female reproductive system [123]. Furthermore, microfluidic chips are revolutionizing the field of assisted reproductive technology, where they are used in in vitro fertilization, embryo culture, embryo screening, and more. Their high integration, precision, and affordability provide new solutions for infertility and other reproductive issues. With the potential for fine sequence control, these chips can sequence the sperm to enter the oocyte in the right direction and time, drastically improving the fertilization rate. Additionally, the introduction of automated equipment means that high-throughput in vitro fertilization and embryo culture can be achieved, thereby maximizing the success rate of reproduction. By enabling ultra-fast embryo screening, microfluidic chips optimize the embryo transfer operation, further enhancing the success rate of assisted reproductive technology.

The challenge of achieving the required functionalities in the future hinges on conducting extensive research that delves into the accuracy, reliability, and stability of microfluidic chip design and manufacturing. Given that microfluidic chips involve complex biological processes such as bacteria and cells, several technical challenges persist in their manufacturing and operation. Currently, the manufacturing technology of microfluidic chips relies on a soft lithography process that utilizes PDMS material. This process is prone to pollution and leakage, while the microstructure and channel of the chip remain complex, causing etching and deposition processes to trap impurities that greatly compromise the overall stability of microfluidic chip operation. The manufacture of the second microfluidic chip requires highly accurate equipment and materials, and the processing process is complex and relatively expensive. Therefore, continuous innovation and optimization of technology are necessary to reduce manufacturing costs and better align with the needs of related bioengineering research. 3D printing demonstrates excellent potential in the preparation of microfluidic chips, which enable achieving high-precision complex microfluidic structures and integrated preparation, thereby reducing the cumbersome process steps. And it has great potential for the development of integrated chips, but more suitable processing materials are essential to address the issue of chip biocompatibility. Additionally, given that the size of microfluidic chips is generally within the range of a few microns to hundreds of microns, fluid flow at the microscale differs significantly from macro-scale flow, necessitating the consideration of microfluid mechanics phenomena such as inertia force and surface tension. It is crucial to ensure such dynamics do not affect cells to avoid non-essential factors, such as cell damage, that would compromise the integrity of the entire process.

**Fig. 8:**
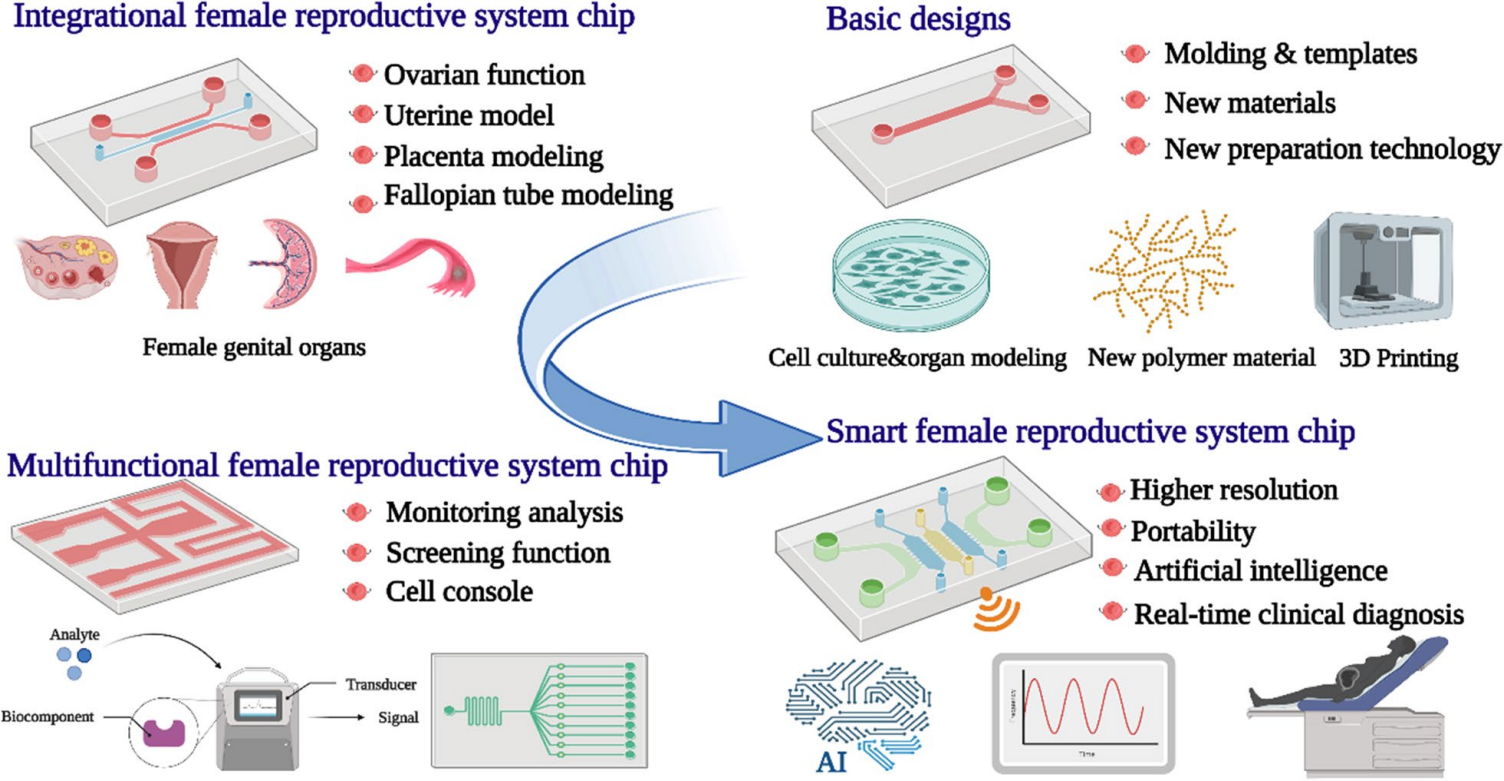
The future of female reproductive system organ chips will develop rapidly from simple cell models to overall organ function models and intelligent clinical chip systems that apply new materials, new technologies, integrated analysis platforms, and artificial intelligence. Created with https://www.biorender.com

In conclusion, despite the vast potential of microfluidic chips in female reproductive health applications, continuous technological development and improvement are necessary to surmount the above challenges and contribute significantly to the development of more efficient and accurate female reproductive health diagnosis and treatment methods (Fig. 8).

## Data Availability

Not applicable.
